# Cuproptosis-related genes associated with mitochondrial dysfunction in Parkinson’s disease

**DOI:** 10.1371/journal.pone.0327550

**Published:** 2025-07-17

**Authors:** Tingting Liu, Jingwen Li, Junshi Zhang, Jianshe Wei

**Affiliations:** Institute for Brain Sciences Research, School of Life Sciences, Henan University, Institute of Neurourology and Urodynamics, Huaihe Hospital of Henan University, Kaifeng, the People’s Republic of China; Jhargram Raj College, INDIA

## Abstract

Parkinson’s disease (PD), a neurodegenerative condition characterized by the loss of dopamine neurons and motor deficits, has recently been associated with cuproptosis, a process potentially leading to mitochondrial dysfunction. This study utilized six PD datasets from the GEO database, designating one for internal training and the remaining five for external validation. Various analytical methods, such as Gene Set Enrichment Analysis (GSEA), immune infiltration studies, and differential expression analysis, were employed to pinpoint differentially expressed genes (DEGs). The research also applied Weighted Gene Co-expression Network Analysis (WGCNA) to identify module genes, followed by Gene Ontology (GO) and Kyoto Encyclopedia of Genes and Genomes (KEGG) enrichment analyses. By intersecting DEGs with cuproptosis-related genes (CRGs), differentially expressed cuproptosis-related genes (DECRGs) were identified and assessed using Receiver Operating Characteristic (ROC) curves. Further analysis led to the discovery of differentially expressed cuproptosis-mitochondrial dysfunction-related genes (DEC-MDRGs), which were validated and subjected to additional scrutiny. The study concluded with predictions of potential therapeutic drugs. The findings revealed 6685 DEGs and 31 distinct modules, with gene functions predominantly enriched in immune-related pathways. Twelve DECRGs, recognized as high-diagnostic-potential hub genes (AUC > 0.9), were identified for early PD diagnosis. Additionally, eight DEC-MDRGs were found to be expressed across various neural cells. The miRNA network highlighted the significance of miR-4632 and miR-637. In a MPTP-induced mouse model of PD, differential gene expression was confirmed through gene and protein analysis. Transmission electron microscopy (TEM) uncovered mitochondrial alterations in SH-SY5Y cells. Potential PD treatments, including NADH, Radicipol, and Glycine, were also identified. In summary, advancements in PD prevention, diagnosis, and treatment can be achieved by modulating copper metabolism and mitochondrial function, thereby enhancing the quality of life for patients.

## Introduction

Parkinson’s disease (PD) is a neurodegenerative condition primarily impacting the substantia nigra (SN), a critical brain region responsible for dopamine production, a neurotransmitter essential for motor control [1]. The gradual loss of dopamine-producing neurons leads to a range of motor and non-motor symptoms, such as tremors, muscle rigidity, bradykinesia (slowed movement), and cognitive decline [2]. The development of PD is shaped by a combination of genetic and environmental factors. A hallmark of PD pathology is the selective degeneration of dopamine neurons in the SN, along with the formation of Lewy bodies (LBs), which are intracellular aggregates rich in α-synuclein (α-syn). The progressive death of SN neurons is a key driver of PD, significantly impairing patients’ motor abilities and overall quality of life [3,4].

Metal ions play a vital role in human health, but their unregulated use, coupled with environmental and agricultural influences, has contributed to a rising incidence of PD. The causes of PD are multifaceted, and the relationship between common metal ions and the disease is significant [5]. Metal ions accumulate in the brain by boosting metabolic activity and eliminating damaged pathways, leading to increased α-syn levels, oxidative stress, and dopamine neuron degeneration, thereby initiating PD. Recent research has highlighted a strong connection between copper accumulation, its toxicity, and PD [6]. Excessive copper buildup can destroy SN neurons, accelerating disease progression. Mutations in specific genes, such as those encoding copper transporters, are thought to be a key mechanism behind copper-induced cell death in PD [7]. Elevated copper levels have been detected in the cerebrospinal fluid and SN of PD patients, and copper has been shown to bind tightly to soluble α-syn, promoting its aggregation [8].

The expression of α-syn in dopamine neurons varies with changes in copper ion levels. Increased copper levels in the body lead to a significant rise in reactive oxygen species (ROS) within cells. When ROS levels surpass the body’s antioxidant capacity, oxidative stress occurs, contributing to PD development [9]. Copper ions are essential for maintaining mitochondrial function, but both excess and deficiency can disrupt mitochondrial activity [10]. At high concentrations, copper ions may bind to mitochondrial proteins, causing oxidation and functional impairment. This disrupts the mitochondrial respiratory chain and oxidative phosphorylation, affecting energy production and adenosine triphosphate (ATP) synthesis, ultimately leading to cellular energy deficits and mitochondrial dysfunction. Conversely, copper deficiency can reduce the activity of mitochondrial cytochrome C oxidase and superoxide dismutase (SOD) [11], impairing mitochondrial energy metabolism and the electron transport chain (ETC).

Copper plays a critical role as a component of dopamine β-hydroxylase, contributing to the biosynthesis of catecholamines [12]. It also holds a unique position in regulating axonal growth, excitotoxic cell death, and synaptic plasticity within the central nervous system (CNS) [13]. Furthermore, copper ions are intricately connected to the mitochondrial antioxidant system. As mitochondria serve as the primary sites of oxidative stress in cells, copper ions function as essential co-factors for various antioxidant enzymes. Elevated levels of copper ions have been observed in the brains of PD patients, potentially linked to increased oxidative stress, mitochondrial dysfunction, and neuronal damage. High copper concentrations may also exacerbate dopamine neuron toxicity, potentially triggering or worsening PD symptoms [8,14]. Conversely, other studies suggest that copper deficiency could also play a role in the onset and progression of PD [15,16]. Thus, both excessive and insufficient copper levels can disrupt mitochondrial antioxidant balance, leading to oxidative damage and impaired mitochondrial function.

In this study, we began by analyzing differentially expressed genes (DEGs) in peripheral blood mononuclear cells (PBMCs) of PD patients using data from the GEO database (https://www.ncbi.nlm.nih.gov/). We then performed an intersection analysis of these DEGs with cuproptosis-related genes (CRGs) and mitochondrial dysfunction-related genes (MDRGs). Using receiver operating characteristic (ROC) curve analysis, we identified DLAT, DLD, FDX1, PDHA1, PDHB, GLRX5, HSPA1A, and LIAS as having strong diagnostic potential, suggesting their utility as biomarkers for PD treatment. Our results revealed that these proteins are highly expressed in the brain, with significant variations in their levels between infancy and adulthood. Additionally, microRNAs (miRNAs) such as miR-4632 and miR-637 were found to play important roles in PD pathogenesis and are associated with these proteins. We also predicted several potential therapeutic agents for PD, including NADH, Radicipol, and Glycine. By deepening our understanding of cuproptosis in PD, we highlighted the importance of maintaining copper ion homeostasis. Significant deviations in copper levels can lead to neurotoxic effects, and elucidating the precise mechanisms by which copper ions influence PD development may advance treatment strategies involving essential and heavy metals. This could also provide new potential targets for the prevention and treatment of PD.

## Materials and methods

### Data acquisition

**Fig 1:**
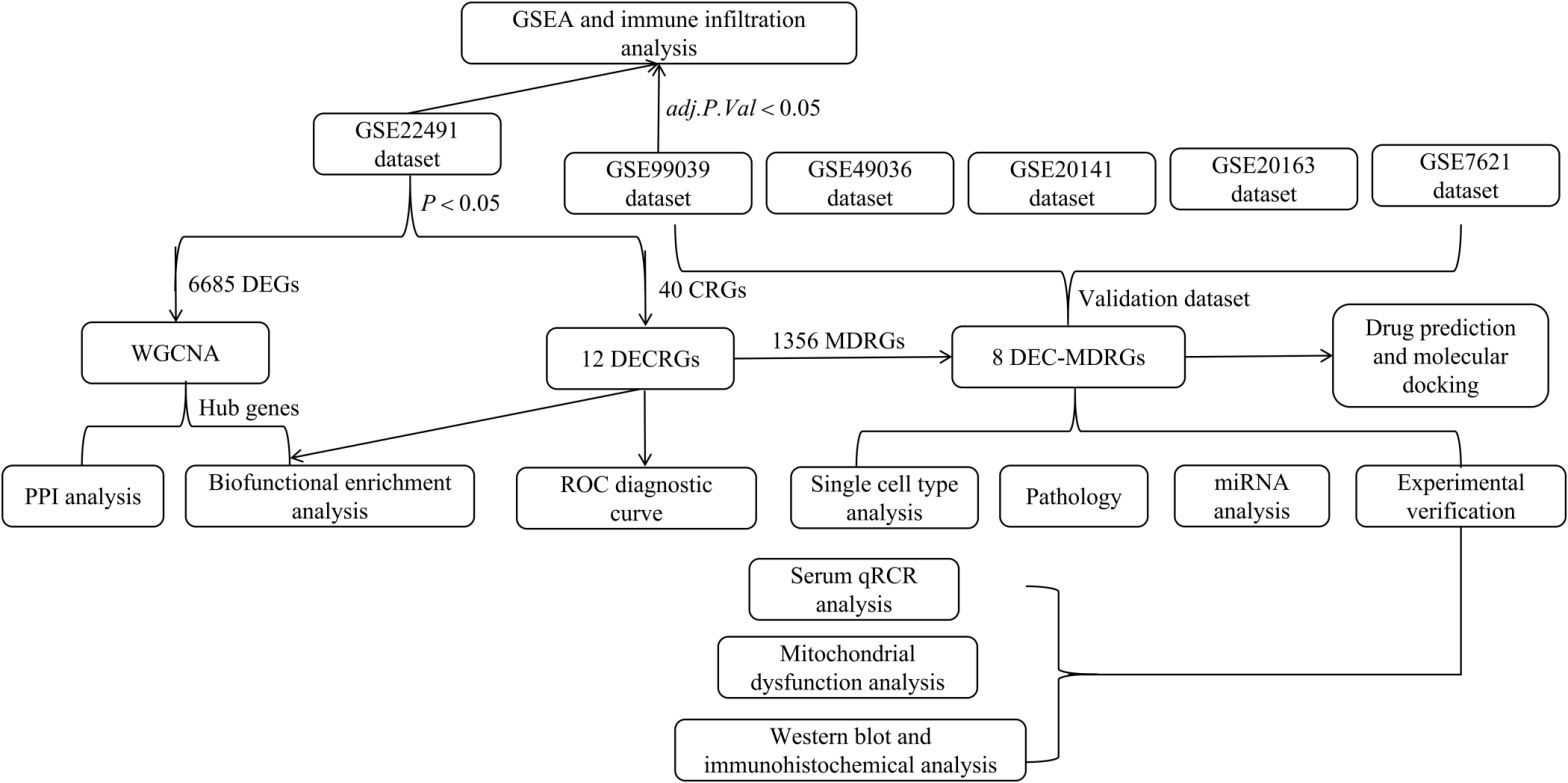
The flow chart of this study. GSEA, Gene set enrichment analysis; DEGs, diferentially expressed genes; WGCNA, Weighted correlation network analysis; PPI, Protein-protein interaction; CRGs, Cuproptosis-related genes; DECRGs, differentially expressed cuproptosis-related genes; MDRGs, mitochondrial dysfunction-related genes; DEC-MDRGs, differentially expressed cuproptosis-mitochondrial dysfunction-related genes; ROC, Receiver operating characteristic.

We obtained microarray and RNA-seq datasets from the GEO database [17], including GSE22491 (Agilent-014850 Whole Human Genome Microarray), GSE99039, GSE20141, GSE20163 (Affymetrix Human Genome U133 arrays), GSE7621, and GSE49036. These datasets were derived from diverse sample types and platforms: GSE22491 contained peripheral blood mononuclear cells (PBMCs) from 10 PD patients and 8 healthy controls; GSE99039 included whole blood samples from 205 idiopathic PD (IPD) patients and 233 controls; GSE20141 and GSE20163 consisted of brain tissue from PD patients and controls, with samples originating from substantia nigra pars compacta (SNpc) neurons and postmortem brains, respectively; GSE7621 also utilized postmortem brain tissue from 16 PD patients and 9 controls; and GSE49036 analyzed transcriptomic data from postmortem SN tissues of individuals with incidental Lewy body disease (iLBD), PD donors, and controls categorized by Braak α-syn staging. For RNA-seq data analysis, we employed DESeq2 (v1.43.1), limma, and GEO2R, with differential gene expression visualized using MD plots and sample relationships depicted through Uniform Manifold Approximation and Projection (UMAP) dimensionality reduction. The overall workflow is summarized in Fig 1.

### GSEA and immune infiltration analysis.

Gene Set Enrichment Analysis (GSEA) assesses the distribution of genes within predefined gene sets obtained from the MSigDB database (https://www.gsea-msigdb.org/gsea/msigdb). It compares these gene sets against a ranked list of genes based on a specific phenotype to determine their relevance and association with that phenotype. We conducted GSEA using the clusterProfiler [4.4.4] package in R (version 4.2.1). Immune infiltration analysis leverages transcriptomic or expression profile data, applying computational algorithms to estimate the abundance of immune cells within tissues, thereby deducing their immune cell composition. For our dataset, we employed the ssGSEA algorithm from the GSVA R package [18] to calculate immune infiltration scores, utilizing markers for 24 immune cells as outlined in the Immunity journal [19].

### Weighted correlation network analysis (WGCNA) [20]

For WGCNA, gene expression data from microarray or RNA-seq is preprocessed by selecting relevant samples and filtering out genes with low information content. The data is normalized, log-transformed, and genes with low variance are removed. A Pearson correlation matrix is subsequently generated to capture gene-gene relationships. To minimize noise, a power function is applied to transform this matrix into an adjacency matrix, emphasizing strong correlations while downplaying weaker ones. Using predefined threshold parameters, the adjacency matrix is used to construct a gene co-expression network, often employing soft thresholding to achieve an optimal balance between network sparsity and connectivity. The network is then divided into modules, which are clusters of highly interconnected genes, through hierarchical clustering or dynamic tree-cutting methods. The significance and robustness of these modules are evaluated by assessing their preservation across datasets and their association with known phenotypes or biological functions. Hub genes, which are highly connected and play a central role in the phenotype, are identified by correlating gene expression with external traits. Finally, heatmaps, gene ontology, and pathway enrichment analyses are used to uncover the biological mechanisms and pathways associated with these modules and hub genes.

### DEGs analysis

Protein-protein interaction (PPI) analysis of DEGs was performed using the String database [21]. Hub genes were identified and analyzed using Cytoscape v3.9.1 with the cytohubba plugin, while functional enrichment analysis, including Kyoto Encyclopedia of Genes and Genomes (KEGG) pathways and Gene Ontology (GO) terms, was carried out using the “clusterProfiler” [4.4.4] and “GOplot” packages in R (version 4.2.1). CRGs were obtained from the ferrdb database [22], and their overlap with DEGs, referred to as differentially expressed cuproptosis-related genes (DECRGs), was visualized using the “venneuler” package in R 4.2.0. DECRGs were further annotated for biological functions using GeneCards [23]. To screen for diagnostic genes, receiver operating characteristic (ROC) curves and area under the curve (AUC) values were calculated using the pROC package in R (4.2.1) to assess the predictive performance of DECRGs.

### Analysis of MDRGs

The overlap between DECRGs and MDRGs, termed differentially expressed cuproptosis-mitochondrial dysfunction-related genes (DEC-MDRGs), was depicted using the “venneuler” package in R 4.2.0. Expression levels of DEC-MDRGs were visualized using the GSE49036 dataset. Protein localization, pathology, and single-cell type analyses were retrieved from Human Protein Atlas, Gene4PD, and Human Transcriptome Cell Atlas. Interactions between diagnostic genes and miRNAs were predicted using the miRCode database and visualized in Cytoscape v3.9.1.

### Experiment validation

#### Animal.

Male C57BL/6J mice, aged six to eight weeks and weighing between 25 and 30 grams, were maintained in a controlled environment with a 12-hour light/dark cycle, a temperature of 22 ± 2°C, and humidity levels of 55 ± 5%. They had unrestricted access to food and water throughout the study. All experimental protocols involving animals adhered to ethical standards and received approval by the Ethics Committee of Henan University (No. HUSOM2021−161). Mice were anesthetized via intraperitoneal injection of sodium pentobarbital (50 mg/kg) (Germany, Merck) prior to all surgical procedures. For sacrifice, mice were deeply anesthetized with sodium pentobarbital (100 mg/kg) and euthanized by cervical dislocation to minimize suffering. Researchers were unaware of the group assignments to ensure unbiased results. During the experimental period, animal health and behavior were monitored daily by trained personnel. Humane endpoints were strictly followed, and any animal exhibiting severe distress (e.g., prolonged immobility, labored breathing, or inability to access food/water) was euthanized immediately. Following a two-week adaptation period, the mice were randomly assigned to two groups, each consisting of eight individuals: the Saline group and the MPTP-treated model group, which received daily intraperitoneal injections of MPTP (20 mg/kg) for 15 days.

#### Serum qPCR analysis.

Collect serum samples from mice (Saline group and MPTP group) and centrifuge at 4000 rpm for 10 min. Store the serum samples at −80°C to avoid RNA degradation. Extract total RNA from the serum samples using an RNA extraction kit (Beyotime, R0091S, China) and assess the purity. Transcribe the extracted total RNA into cDNA using a reverse transcription kit (Thermo Fisher, K1622, USA). Perform PCR amplification (SYBR Green PCR Master Mix, Toyobo, QPK-201, Japan) (95 °C, 30s; 40 cycles, 95 °C, 15s, 60 °C, 30s; collect fluorescence signals every 0.5 °C increase in temperature) and analyze the fluorescence signals using a real-time PCR machine. The fluorescence curves of the reactions can be converted into expression levels of corresponding genes and subjected to relative quantification analysis with a reference gene or control group. The primer sequence is shown in **Table 1**.

**Table 1:** Primer sequence.

Gene	Species	Primer sequence (5’-3’)	
***DLAT***	Mouse	Forward primer	5’-AGGGTGTTCGTTAGTCCTCTTGC-3’
		Reverse primer	5’-TCTTGATGATTCTGCCTTCTGG-3’
***DLD***	Mouse	Forward primer	5’-TGCTATTGGAGATGTGGTCGCT-3’
		Reverse primer	5’-TACCCTCTTCTTTCAACTGTTCTTC-3’
***FDX1***	Mouse	Forward primer	5’- ACAGACAGGAACCTGGAAGACC-3’
		Reverse primer	5’- GAGACAATCTGTATGGGGTGGTT-3’
***PDHA1***	Mouse	Forward primer	5’-ACCAGAGAGGATGGGCTCAAGT-3’
		Reverse primer	5’-AGGTGGTCCGTAGGGTTTATGC-3’
***PDHB***	Mouse	Forward primer	5’-AAGAAGTTGCCCAGTATGACGGT-3’
		Reverse primer	5’-GCTTGCATAGAGAAATTGAAGGTCA-3’
***GLRX5***	Mouse	Reverse primer	5’-GACAAGGTGGTGGTGTTCCTC-3’
		Forward primer	5’-TTGAGGTACACTTGCGGGATG-3’
***HSPA1A***	Mouse	Reverse primer	5’-AGGCTGACAAGAAGAAGGTGCTG-3’
		Forward primer	5’-CCTGGTACAGCCCACTGATGAT-3’
***LIAS***	Mouse	Reverse primer	5’-GCTGAGCACATCGCCAAGAC-3’
		Forward primer	5’-TCCAGACAGAGCCACCTTCTC-3’
***β-actin***	Mouse	Reverse primer	5’-GTGACGTTGACATCCGTAAAGA-3’
		Forward primer	5’-GTAACAGTCCGCCTAGAAGCAC-3’

#### Western blot and Immunohistochemical.

SN tissue was homogenized in ice-cold RIPA buffer with PMSF (Servicebio, China) using a Polytron, followed by sonication and centrifugation at 12,000 × g for 10 minutes at 4°C. Protein concentration in the supernatant was assessed by BCA assay, separated via SDS-PAGE, and transferred to a nitrocellulose membrane (Millipore, Germany). The membranes were blocked with 5% nonfat milk in TBST for 1 hour at room temperature and then incubated overnight at 4°C with primary antibodies against DLAT, DLD, FDX1, PDHA1, PDHB, GLRX5, HSPA1A, LIAS, DLST, Lipoic Acid, and GAPDH (Affinity, Melbourne; Abcam, Britain) diluted in TBST with 1% nonfat milk. After washing, the membranes were incubated with HRP-conjugated secondary antibody for 2 hours at room temperature and developed using an Enhanced Chemiluminescence assay (BIO-Rad).

Immunohistochemistry was performed on 25 µm thick brain sections. Antigen retrieval was done using 1xCitrate solution at 98°C for 10 minutes, followed by permeabilization with 0.1% Triton X-100 for 10 minutes, hydrogen peroxide treatment for 20 minutes in the dark, and blocking with 10% goat serum in PBS. Sections were then incubated overnight at 4°C with primary antibodies against DLAT, DLD, FDX1, PDHA1, PDHB, GLRX5, HSPA1A, and LIAS (Affinity, Melbourne). After washing, sections were treated with appropriate biotin secondary antibody and visualized using a DAB Reagent Kit (Servicebio, China). Densitometry analysis of Western blot and immunohistochemistry images was conducted using ImageJ software (https://imagej.net/software/imagej/). Protein expression levels were normalized to β-actin as a loading control, and ratios of target protein to β-actin were calculated for statistical analysis.

#### Observing mitochondrial structure using transmission electron microscopy (TEM).

Sectioning and electron microscopy fixation of mouse brain SN tissue were performed as follows: The tissue was fixed with electron microscopy fixation solution (G1102, Servicebio, China) at room temperature for 2 h and then stored at 4°C in the dark to maintain the integrity of its cellular structure. The resin-embedded SN tissue samples were prepared into transparent ultrathin sections using a microtome, with a thickness of approximately 70–100 nm. The sections were dehydrated with gradient ethanol-acetone. Negative staining was performed using a negative staining solution containing 2% phosphotungstic acid. The negatively stained sections were observed for the morphology and location of mitochondria using a TEM.

#### Detection of cellular ROS and mitochondrial membrane potential.

SH-SY5Y cells were grown in DMEM medium with 10% FBS in a 37°C incubator with 5% CO_2_. When cells reached 90% confluence, they were seeded into a 24-well plate and divided into a control group and an mpp^+^ (100μM) group, both cultured for 24 hours. For ROS detection, cells were incubated with 300 μL of a 1:1000 diluted DCFH-DA probe (G1706, Servicebio) for 30 minutes, followed by three PBS washes. Mitochondrial membrane potential was assessed using the JC-1 Kit (G1515, Servicebio), with cells stained for another 30 minutes and then washed three times with PBS. In MPTP-induced PD mice, mitochondria from the SN were isolated using a kit (C3606, Beyotime). Fluorescence intensity was observed under a microscope and analyzed with ImageJ software.

### Drug prediction and molecular docking.

Predict protein-related therapeutic drug using the Drugbank database (https://go.drugbank.com/) [24]. Download the drug SDF format file and protein PDB format file. Perform molecular docking using Discovery Studio (DS) software to analyze the binding site and binding affinity between the protein and the drug.

### Statistical analysis.

Using Graphpad Prism for statistical analysis of data, the data is represented by mean ± SEM, and comparisons between groups were performed using an unpaired Student’s t−test. ROCs were used to evaluate AUCs and predictive abilities. A *pValue* of less than 0.05 was considered statistically significant.

## Results

### Differential genes acquisition

**Fig 2:**
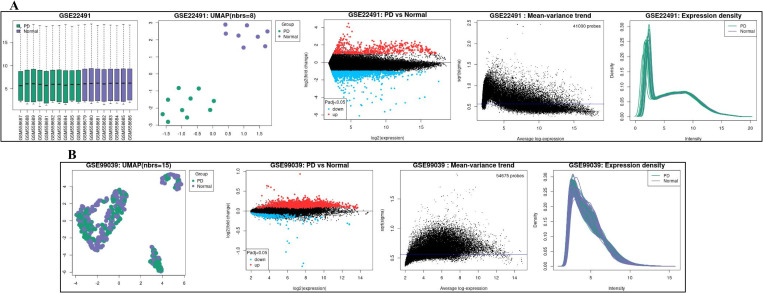
Gene chip data information. (A) Chip data of diferential genes in Parkinson’s disease compared with the healthy controls of GSE22491 dataset. (B) Chip data of diferential genes in Parkinson’s disease compared with the healthy controls of GSE99039 dataset.

In the GSE22491 dataset, a notable distinction was observed between 10 PD patients and 8 healthy controls in peripheral blood mononuclear cells, revealing a total of 6685 differentially expressed genes (*P* < 0.05). Similarly, analysis of the GSE99039 dataset, which included 233 healthy controls and 205 PD patients using whole blood samples, identified 1148 differentially expressed genes (*adj.P.Val *< 0.05). UMAP and mean-variance trend analysis demonstrated that the data exhibited reproducibility and lacked significant trends, while boxplot distributions displayed consistent linear patterns. Expression density plots were used alongside boxplots to verify data normalization prior to conducting differential expression analysis (Figs 2A and 2B).

### GSEA and immune infiltration analysis

**Fig 3:**
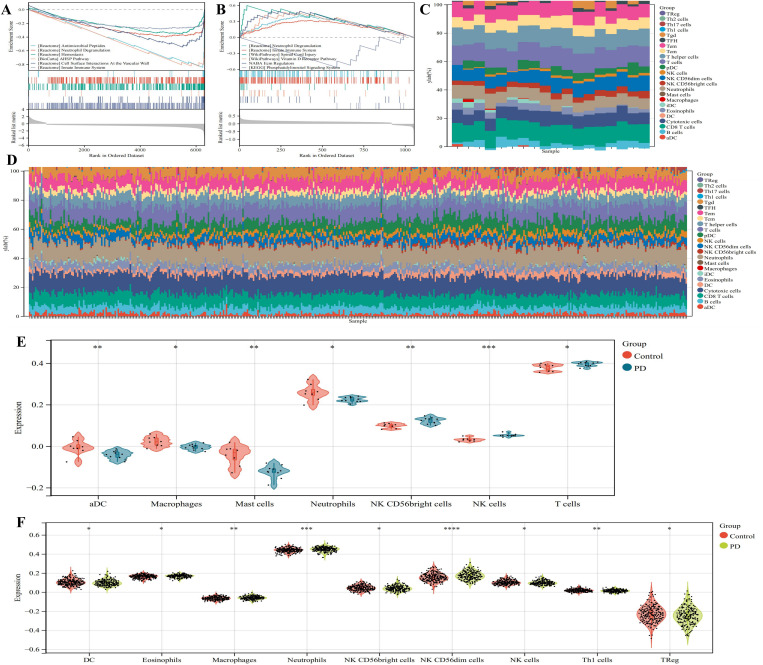
GSEA and immune infiltration analysis. (A) GSEA enrichment analysis of the GSE22491 dataset. (B) GSEA enrichment analysis of the GSE99039 dataset. (C) Heatmap of immune infltration analysis of diferential genes in the GSE22491 dataset. (D) Heatmap of immune infltration analysis of diferential genes in the GSE99039 dataset. (E) Boxplot of diferentially expressed immune cells in the GSE22491 dataset. (F) Boxplot of diferentially expressed immune cells in the GSE99039 dataset. Compared with healthy controls, *P < 0.05, **P < 0.01, ***P < 0.001.

In the GSE22491 dataset, GSEA revealed that the differentially expressed genes were primarily associated with antimicrobial peptides, neutrophil degranulation, hemostasis, the biocarta AHSP pathway, cell surface interactions at the vascular wall, and the innate immune system (Fig 3A). Similarly, in the GSE99039 dataset, GSEA indicated that the differential genes were mainly involved in neutrophil degranulation, the innate immune system, spinal cord injury, the vitamin D receptor pathway, ECM regulators, and the phosphatidylinositol signaling system (Fig 3B). Heatmaps illustrating immune infiltration for the GSE22491 and GSE99039 datasets are presented in Figs 3C and 3D, respectively. In the GSE22491 dataset, significant differences were observed in aDC, macrophages, mast cells, neutrophils, NK CD56bright cells, NK cells, and T cells. Compared to healthy controls, aDC, macrophages, mast cells, and neutrophils were downregulated in PD, while NK CD56bright cells, NK cells, and T cells were upregulated (Fig 3E). In the GSE99039 dataset, differences were noted in DC, eosinophils, macrophages, neutrophils, NK CD56bright cells, NK CD56dim cells, NK cells, Th1 cells, and TReg. Relative to healthy controls, DC, NK CD56bright cells, NK cells, Th1 cells, and TReg were downregulated in PD, whereas eosinophils, macrophages, neutrophils, and NK CD56dim cells were upregulated (Fig 3F). No significant differences in immune cell expression were observed, as shown in S1 Fig. The role and significance of immune cells in PD treatment are increasingly being recognized and studied. Research has demonstrated that modulating the quantity and function of immune cells can effectively alleviate motor symptoms and neural damage in PD patients. For instance, drug therapies that inhibit the secretion of inflammatory factors by immune cells can reduce motor neuron apoptosis and CNS inflammation, thereby improving PD symptoms. Additionally, cellular therapies, such as stem cell-based approaches to repair damaged neurons or artificial modulation of specific immune cell populations to mitigate inflammation and neuronal damage, are under investigation. These studies offer new perspectives and directions for PD treatment [25,26].

### WGCNA screened DEGs

**Fig 4:**
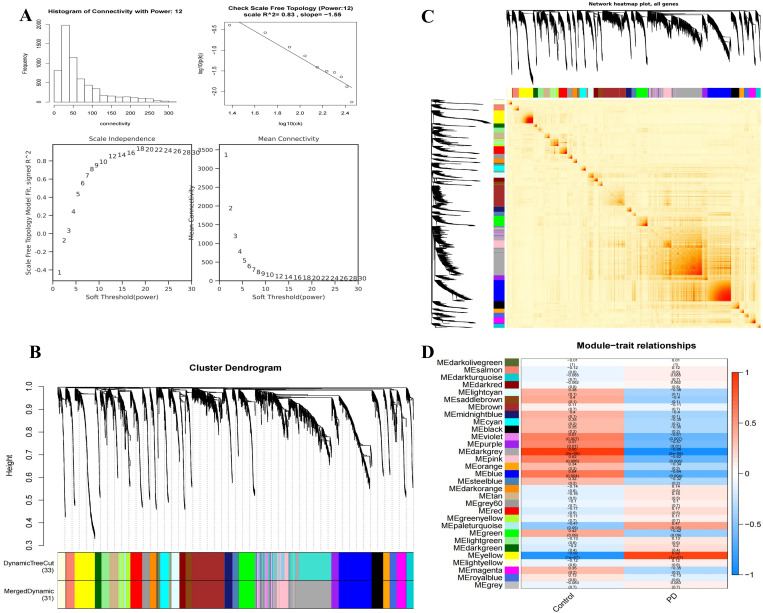
Screening differentially expressed genes in the GSE22491 dataset using WGCNA. (A) WGCNA network building parameters. (B) The upper part of the figure shows the gene clustering tree constructed based on the dissTOM matrix generated by weighted correlation, while the lower part shows the distribution of genes in each module. Genes in the same color belong to the same module. The colors obtained by Dynamic Tree Cut method represent modules identified by this method. Due to certain correlations between some modules, corresponding modules are merged into one, indicated by “Merged Dynamic” at the bottom, which will be used for subsequent analysis. (C) Heatmap of all gene clusters. (D) Heatmap showing the correlation between trait modules.

To achieve a scale-free network distribution, an appropriate value for the adjacency matrix weight parameter, known as power, must be selected. A range of power values from 1 to 30 was tested, and the corresponding correlation coefficients and average connectivity of the resulting networks were computed. Higher correlation coefficients (with a maximum of 1) indicate a network that more closely approximates a scale-free distribution. However, it is also essential to maintain a sufficient level of gene connectivity. Thus, the chosen power value must strike a balance between achieving a high correlation coefficient and ensuring adequate gene connectivity. For this analysis, a power value of 12 was selected (Fig 4A). Using this power value, a weighted gene co-expression network model was constructed, and 6260 genes were ultimately grouped into 31 modules. The grey module, representing genes that could not be assigned to any specific module, was excluded from further analysis as it holds no reference value (Fig 4B). Fig 4C displays the clustering heatmap of all genes. Modules associated with specific traits were identified based on an absolute correlation coefficient ≥0.3 and a p-value <0.05. For each trait-related module, the correlation between module gene expression and the corresponding trait (Gene Significance, GS) was calculated, along with the correlation between module gene expression and the module eigengene. A scatter plot was generated based on these values, demonstrating that the genes within the module exhibited strong correlations with both the traits and the module eigengenes (Fig 4D). Through this analysis, 438 hub genes were identified (S2 Fig).

### Hub genes screened

**Fig 5:**
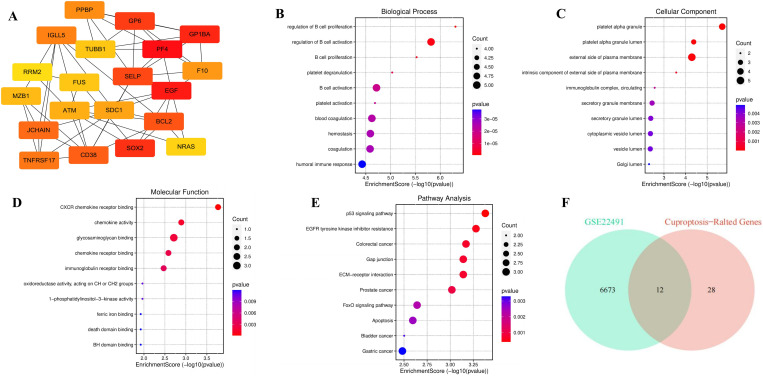
Functional enrichment analysis of hub genes. (A) Hub genes were selected using the MCC algorithm. (B) Enrichment analysis of biological processes (BP) hub genes. (C) Enrichment analysis of cellular components (CC) of hub genes. (D) Enrichment analysis of molecular functions (MF) of hub genes. (E) Enrichment analysis of signaling pathways of hub genes. (F) Venn diagram showing the overlap between hub genes from GSE22491 and cuproptosis-related genes (CRGs).

The STRING database was employed to screen for PPI. The analysis revealed 389 nodes, 466 edges, an average node degree of 2.4, an average local clustering coefficient of 0.363, an expected number of edges of 392, and a PPI enrichment *p*-value of 0.000154 (S3 Fig). Using the CytoHubba plugin in Cytoscape, the top 20 hub genes were identified based on connectivity algorithms (Fig 5A). Genes with higher scores in each algorithm, particularly the MCC algorithm, were selected as hub genes. GO and KEGG analyses were conducted to explore the potential biological functions and enriched pathways of these hub genes. The GO analysis categorized the findings into biological processes (BPs), cellular components (CCs), and molecular functions (MFs). The BP analysis revealed that hub genes were primarily enriched in processes such as regulation of B cell proliferation, B cell activation, platelet degranulation, blood coagulation, hemostasis, and humoral immune response (Fig 5B). The CC analysis indicated enrichment in platelet alpha granule, external side of plasma membrane, immunoglobulin complex, secretory granule membrane, and vesicle lumen (Fig 5C). The MF analysis highlighted enrichment in CXCR chemokine receptor binding, chemokine activity, glycosaminoglycan binding, oxidoreductase activity, and ferric iron binding (Fig 5D). KEGG pathway analysis demonstrated that hub genes were mainly associated with the p53 signaling pathway, EGFR tyrosine kinase inhibitor resistance, colorectal cancer, ECM-receptor interaction, gap junction, FoxO signaling pathway, apoptosis, bladder cancer, and gastric cancer (Fig 5E).

### Analysis of DECRGs

**Fig 6:**
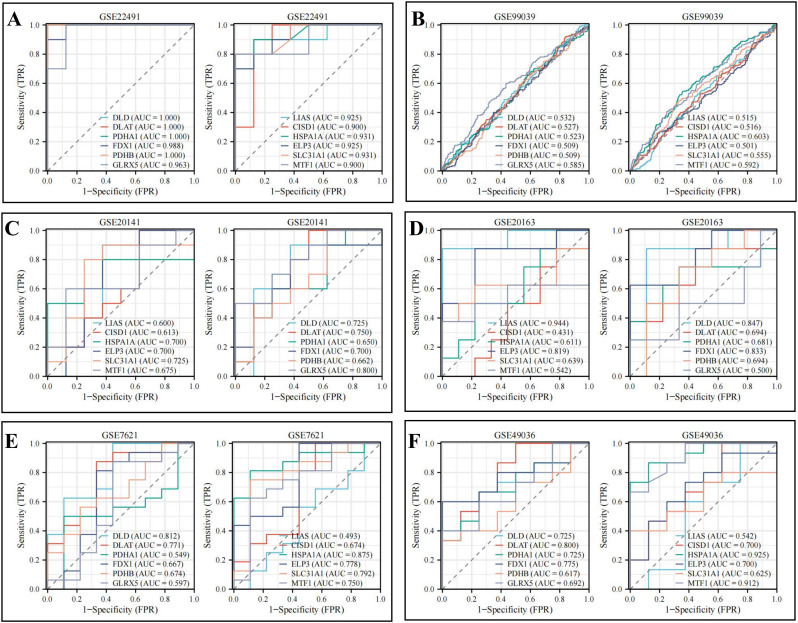
Diagnostic value of 12 hub genes, including (A) GSE22491 dataset, (B) GSE99039 dataset, (C) GSE20141 dataset, (D) GSE20163 dataset, (E) GSE7621 dataset, and (F) GSE49036 dataset.

Finally, by integrating the GSE22491 dataset with CRGs, we identified 12 overlapping genes, referred to as DECRGs, for further investigation (Fig 5F). These genes include DLD, DLAT, PDHA1, FDX1, PDHB, GLRX5, LIAS, CISD1, HSPA1A, ELP3, SLC31A1, and MTF1. The biological functions of these genes are detailed in Table 2, highlighting their involvement in processes such as the tricarboxylic acid (TCA) cycle, acetyl-CoA synthesis and metabolism, and ketone body biosynthesis, all of which influence mitochondrial function. The associated signaling pathways primarily encompass the TCA cycle, pyruvate metabolism, glycolysis/gluconeogenesis, and carbon metabolism (S4 Fig). To assess the diagnostic potential of these 12 hub genes in PD, ROC curves were employed. Fig 6A demonstrates that the 12 hub genes exhibit very high diagnostic value (AUC > 0.9) in the early stages of PD. Further validation using the GSE99039, GSE20141, GSE20163, GSE7621, and GSE49036 datasets confirmed the expression levels and diagnostic utility of these genes (AUC > 0.493) (Figs 6B‒6F). The results indicate that these DECRGs have strong diagnostic value for PD and can help identify the presence or progression of PD based on changes in serum protein levels. Additionally, we explored the correlation between the expression levels of the 12 DECRGs in the GSE22491 dataset, revealing significant associations (S5A Fig).

**Table 2:** Biofunctional annotation of DECRGs.

Gene	Name	Biological function
***DLD***	Dihydrolipoamide dehydrogenase	This gene encodes a member of the class-I pyridine nucleotide-disulfide oxidoreductase family. The encoded protein has been identified as a moonlighting protein based on its ability to perform mechanistically distinct functions. In homodimeric form, the encoded protein functions as a dehydrogenase and is found in several multi-enzyme complexes that regulate energy metabolism. However, as a monomer, this protein can function as a protease. Mutations in this gene have been identified in patients with E3-deficient maple syrup urine disease and lipoamide dehydrogenase deficiency.
***DLAT***	Dihydrolipoamide S-acetyltransferase	This gene encodes component E2 of the multi-enzyme pyruvate dehydrogenase complex (PDC). PDC resides in the inner mitochondrial membrane and catalyzes the conversion of pyruvate to acetyl coenzyme A. The protein product of this gene, dihydrolipoamide acetyltransferase, accepts acetyl groups formed by the oxidative decarboxylation of pyruvate and transfers them to coenzyme A. Dihydrolipoamide acetyltransferase is the antigen for antimitochondrial antibodies.
***PDHA1***	Pyruvate dehydrogenase E1 subunit alpha 1	The pyruvate dehydrogenase (PDH) complex is a nuclear-encoded mitochondrial multienzyme complex that catalyzes the overall conversion of pyruvate to acetyl-CoA and carbon dioxide, and provides the primary link between glycolysis and the TCA cycle. The PDH complex is composed of multiple copies of three enzymatic components: pyruvate dehydrogenase (E1), dihydrolipoamide acetyltransferase (E2) and lipoamide dehydrogenase (E3). The E1 enzyme is a heterotetramer of two alpha and two beta subunits. This gene encodes the E1 alpha 1 subunit containing the E1 active site, and plays a key role in the function of the PDH complex.
***FDX1***	Ferredoxin 1	This gene encodes a small iron-sulfur protein that transfers electrons from NADPH through ferredoxin reductase to mitochondrial cytochrome P450, involved in steroid, vitamin D, and bile acid metabolism. Pseudogenes of this functional gene are found on chromosomes 20 and 21.
***PDHB***	Pyruvate dehydrogenase E1 subunit beta	This gene encodes the E1 beta subunit. Mutations in this gene are associated with pyruvate dehydrogenase E1-beta deficiency.
***GLRX5***	Glutaredoxin 5	This gene encodes a mitochondrial protein, which is evolutionarily conserved. It is involved in the biogenesis of iron-sulfur clusters, which are required for normal iron homeostasis.
***LIAS***	Lipoic Acid Synthetase	The protein encoded by this gene belongs to the biotin and lipoic acid synthetases family. Localized in the mitochondrion, this iron-sulfur enzyme catalyzes the final step in the de novo pathway for the biosynthesis of lipoic acid, a potent antioxidant.
***CISD1***	CDGSH Iron Sulfur Domain 1	This gene encodes a protein with a CDGSH iron-sulfur domain and has been shown to bind a redox-active [2Fe-2S] cluster. The encoded protein has been localized to the outer membrane of mitochondria and is thought to play a role in regulation of oxidation.
***HSPA1A***	Heat shock protein family A (Hsp70) member 1A	This intronless gene encodes a 70kDa heat shock protein which is a member of the heat shock protein 70 family. In conjuction with other heat shock proteins, this protein stabilizes existing proteins against aggregation and mediates the folding of newly translated proteins in the cytosol and in organelles. It is also involved in the ubiquitin-proteasome pathway through interaction with the AU-rich element RNA-binding protein 1. The gene is located in the major histocompatibility complex class III region, in a cluster with two closely related genes which encode similar proteins.
***ELP3***	Elongator Acetyltransferase Complex Subunit 3	Enables acetyltransferase activity and phosphorylase kinase regulator activity. Involved in regulation of transcription by RNA polymerase II and tRNA wobble uridine modification. Located in cytosol and nucleolus. Part of elongator holoenzyme complex.
***SLC31A1***	Solute Carrier Family 31 Member 1	The protein encoded by this gene is a high-affinity copper transporter found in the cell membrane. The encoded protein functions as a homotrimer to effect the uptake of dietary copper.
***MTF1***	Metal Regulatory Transcription Factor 1	This gene encodes a transcription factor that induces expression of metallothioneins and other genes involved in metal homeostasis in response to heavy metals such as cadmium, zinc, copper, and silver. The protein is a nucleocytoplasmic shuttling protein that accumulates in the nucleus upon heavy metal exposure and binds to promoters containing a metal-responsive element (MRE).

### Analysis of DEC-MDRGs

Mitochondria serve as the energy powerhouses of cells, generating the majority of cellular energy. Both copper deficiency and excess can disrupt mitochondrial function, as copper is a vital component of key protein enzymes within mitochondria. These enzymes play essential roles in the normal utilization of cellular metabolites, waste disposal, and critical processes such as oxidation-reduction reactions and energy production. A lack of copper can reduce mitochondrial copper content, impairing enzyme activity and function, and potentially triggering various mitochondrial-related diseases. Consequently, we investigated the expression of mitochondrial dysfunction-related genes in PD. By integrating MDRGs and CRGs, we identified 8 overlapping genes (DEC-MDRGs) for further analysis (S5B Fig). In the GSE22491 dataset, compared to healthy controls, DLAT, DLD, FDX1, PDHA1, PDHB, GLRX5, HSPA1A, and LIAS were all downregulated. However, in the validation datasets, HSPA1A was found to be upregulated (S5C–S5E Figs). These discrepancies may arise from the distinct functions and characteristics of different cell types, which require specific gene expression patterns to meet their unique needs. Additionally, genetic variations and external environmental factors could contribute to these differences. Further experimental studies will be conducted to validate the roles of these DEC-MDRGs.

### Single cell type analysis of DEC-MDRGs

**Fig 7:**
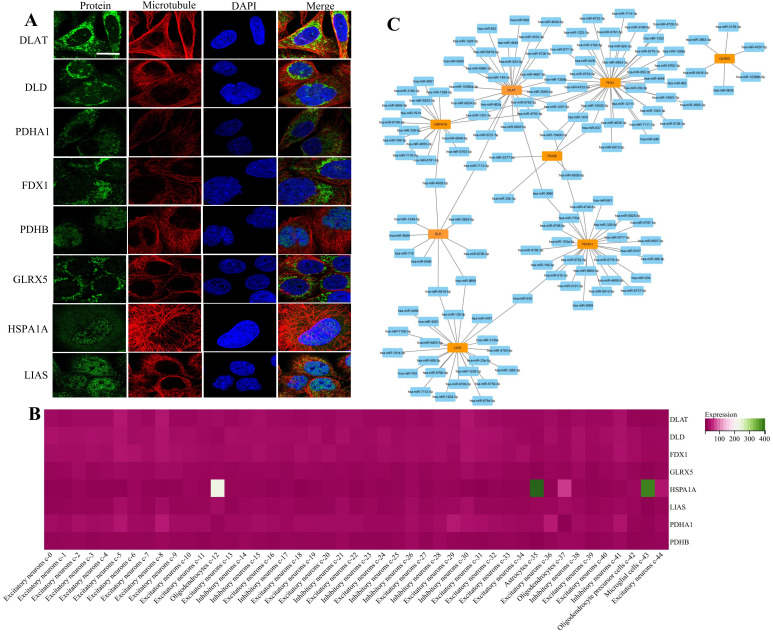
Expression and miRNA analysis of DEC-MDRGs. (A) Location of DLAT, DLD, FDX1, PDHA1, PDHB, GLRX5, HSPA1A, and LIAS proteins in cells: green represents the target protein, red represents microtubules, and blue represents the nucleus (Scale bar, 10 µm). (B) Single cell type was used to analyze the expression of DEC-MDRGs in the brain. (C) Coexpression network of DEC-MDRGs and target miRNAs. Orange rectangle represent DEC-MDRGs, blue rectangle represent target miRNAs.

**Fig 8:**
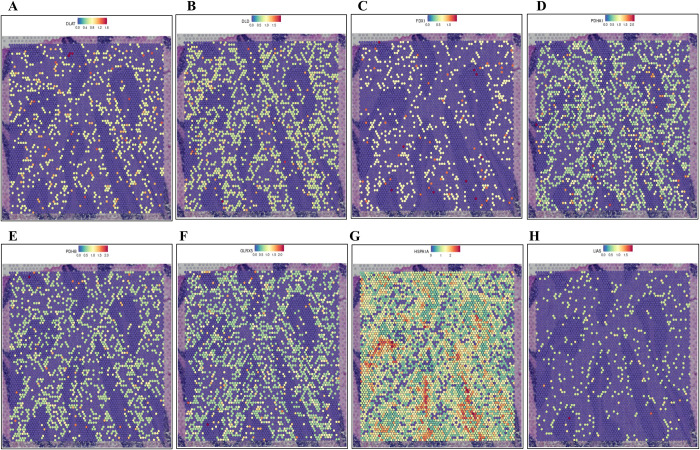
The distribution of proteins in the cerebellum, including (A) DLAT, (B) DLD, (C) FDX1, (D) PDHA1, (E) PDHB, (F) GLRX5, (G) HSPA1A, and (H) LIAS. The redder the color, the higher the level of expression.

Proteins that modulate mitochondrial function are crucial in the treatment of PD. By regulating the expression or activity of these proteins, mitochondrial energy production can be enhanced, oxidative stress reduced, and neuronal survival promoted. Investigating and modulating the distribution of mitochondrial functional proteins in the brain offers new therapeutic opportunities for PD. Further research into the functions and mechanisms of these proteins may yield important insights for developing PD treatment strategies and drugs, potentially opening new avenues for protecting and enhancing neuronal function in the brain [27,28]. To elucidate protein localization, we utilized the HPA database for protein expression visualization and pathological analysis (Fig 7A, S6 Fig). We also examined the expression of DEC-MDRGs in the brain, revealing their enrichment in various neuronal cells, oligodendrocytes, astrocytes, oligodendrocyte precursor cells, and microglial cells (Fig 7B). Additionally, we analyzed the average expression levels of DLAT, DLD, GLRX5, and HSPA1A across different brain regions at various developmental stages, finding that HSPA1A exhibited significant temporal expression differences (S7 Fig). PD patients often exhibit symptoms of cerebellar motor dysfunction, such as postural instability, gait disturbances, and impaired hand coordination, which may result from cerebellar damage. Research indicates that cerebellar function is often compromised in PD patients. The cerebellum, a key structure for motor control and coordination, plays a vital role in precise movement regulation. In PD, the cerebellum may undergo degenerative changes and neuronal damage, leading to diminished motor regulatory function [29,30]. Consequently, we analyzed protein distribution in the cerebellum, revealing high expression of DLD, PDHA1, GLRX5, and HSPA1A in this region (Fig 8). We also explored differences in protein expression across brain cell types during infancy and adulthood (S8 Fig). The cell types and protein expression in the brain vary between infancy and adulthood due to developmental changes such as cell proliferation, migration, differentiation, and the formation of neural connectivity and plasticity [31–33].

### miRNA analysis

The expression of miRNAs associated with the genes is depicted in Fig 7C, with hsa-miR-4632-5p, hsa-miR-6819-5p, hsa-miR-7113-3p, hsa-miR-9899, hsa-miR-1972, hsa-miR-1268b, hsa-miR-1207-5p, hsa-miR-6782-3p, hsa-miR-637, hsa-miR-4750-3p, hsa-miR-638, and hsa-miR-3960 playing significant roles in the miRNA regulatory network. Research has shown that miR-4632 is involved in modulating biological processes and pathways related to inflammation, oxidative stress, mitochondrial function, and neurodegeneration [34]. Similarly, miR-637 has been found to regulate neuroinflammation, mitochondrial function, and neuronal survival [35]. Dysregulation of these miRNAs may contribute to pathological mechanisms in PD, such as dopamine neuron damage, apoptosis, inflammation, and mitochondrial dysfunction. Further investigation into these miRNAs will enhance our understanding of their specific roles in PD and may offer new targeted therapeutic approaches for the disease. Experimental validation is required to confirm these findings.

### Blood serum qPCR analysis

**Fig 9:**
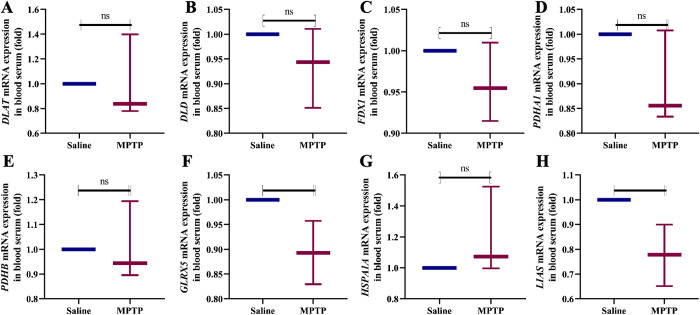
The expression of mitochondrial dysfunction related genes in the blood serum of MPTP induced Parkinson’s disease mice model. (A) *DLAT* mRNA expression. (B) *DLD* mRNA expression. (C) *FDX1* mRNA expression. (D) *PDHA1* mRNA expression. (E) *PDHB* mRNA expression. (F) *GLRX5* mRNA expression. (G) *HSPA1A* mRNA expression. (H) *LIAS* mRNA expression. Compared with the Saline group, “ns” represents no statistical difference, *P < 0.05. n = 3.

The differentially genes identified in this experiment were screened using the GSE22491 dataset, which was sourced from PBMC. To validate the reliability and accuracy of the screening results, mice blood serum was used to detect the target genes. The results showed that, compared with the Saline group, the MPTP group exhibited decreased expression of *DLAT, DLD, PDHA1, FDX1, PDHB, GLRX5*, and *LIAS*, while *HSPA1A* expression was increased (Fig 9). Although changes in gene expression occurred in the MPTP group, most of the genes did not show statistical differences, possibly due to a small sample size, where even if some differences existed, they may not have reached statistical significance. However, these genes still play an important role in regulating mitochondrial function.

### Mitochondrial dysfunction analysis

**Fig 10:**
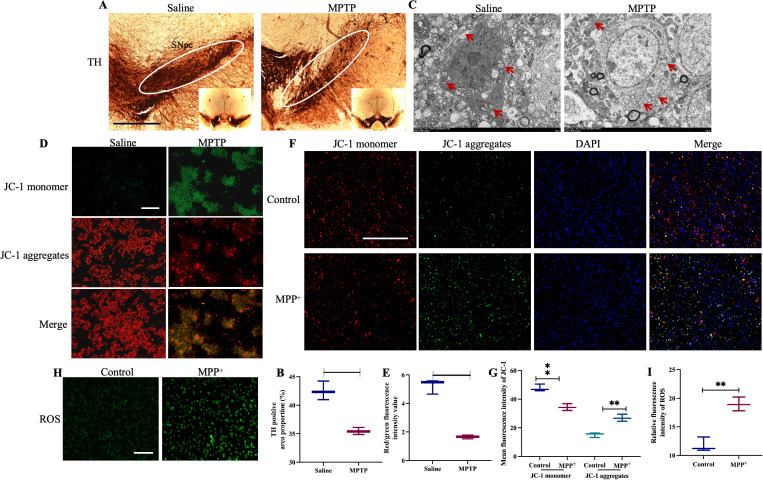
The mitochondrial morphology and function analysis. (A) Immunohistochemical analysis of TH in MPTP-induced PD mice model in vivo. Serious loss of TH in SNpc. (B) Immunohistochemical analysis of TH positive area proportion statistics. (C) Observation of mitochondrial structure by transmission electron microscopy. (D) Detection of mitochondrial membrane potential in the SN of MPTP induced PD mice model using JC-1 mitochondrial membrane potential fluorescent probe staining. (E) JC-1 mean fluorescence intensity statistics. (F) JC-1 mitochondrial membrane potential fluorescent probe staining for MPP^+^ induced SH-SY5Y cells in vitro PD model. (G) JC-1 mean fluorescence intensity statistics. (H) Detection of ROS in Parkinson’s disease model induced by MPP^+^ in SH-SY5Y cells in vitro. (I) ROS relative fluorescence intensity statistics. Compared with the Saline group, **P < 0.01. n = 3.

Tyrosine hydroxylase (TH) is a pivotal enzyme in dopamine synthesis, playing an essential role in the production of dopamine in vivo. Alterations in TH activity can influence dopamine synthesis and release, making it closely associated with PD. Immunohistochemical analysis of TH expression revealed a significant loss of TH in the SN of MPTP-induced mice compared to the Saline group (Figs 10A and 10B). TEM showed a reduction in mitochondrial numbers, disorganized and decreased mitochondrial cristae, increased cristae density, damage to the inner and outer mitochondrial membranes, widespread mitochondrial shrinkage, and the presence of larger vacuoles in the SN of MPTP-induced mice (Fig 10C). Mitochondrial dysfunction is closely linked to changes in mitochondrial membrane potential, often resulting in its reduction or loss. Under normal conditions, the mitochondrial inner membrane maintains a high negative potential, causing JC-1 to aggregate and emit red fluorescence. However, in MPTP- or MPP + -treated mice or SH-SY5Y cells, a decrease in mitochondrial membrane potential prevents JC-1 aggregation, leading to the formation of monomers that emit green fluorescence (Figs 10D–10G). ROS play a significant role in PD by contributing to oxidative stress, mitochondrial dysfunction, and inflammatory responses, thereby participating in the progression of PD and neuronal degeneration. Measurement of ROS levels in SH-SY5Y cells following MPP+ treatment demonstrated a marked increase in ROS content (Figs 10H and 10I).

### Western blot and immunohistochemical analysis

**Fig 11:**
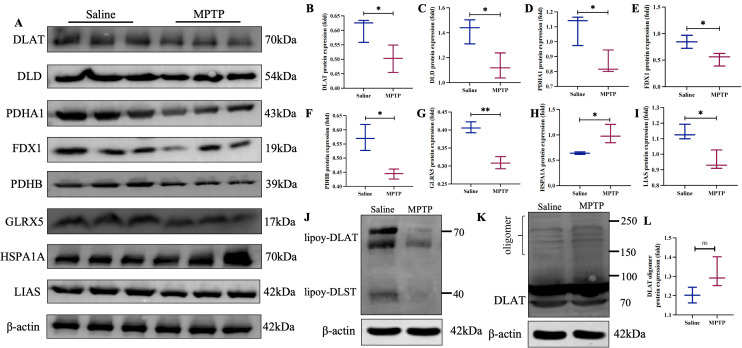
The expression of mitochondrial dysfunction-related proteins in the MPTP-induced mice model in vivo of PD. (A) Each protein strip graph. (B) DLAT protein expression. (C) DLD protein expression. (D) PDHA1 protein expression. (E) FDX1 protein expression. (F) PDHB protein expression. (G) GLRX5 protein expression. **(H)** HSPA1A protein expression. (I) LIAS protein expression. (J) The effect of specific antibodies against lipoic acid on the lipoylation of DLAT and DLST. (K) In the MPTP-induced PD mice model, DLAT undergoes oligomerization and protein oligomers appear. (L) DLAT oligomerization protein expression. Protein expression data are normalized to β-actin. Compared with the Saline group, *P < 0.05, **P < 0.01. ns = no significance. n = 3.

To confirm the expression of mitochondrial dysfunction-related proteins in PD, we conducted western blot and immunohistochemistry analyses (Figs 11A–11I, S9 Fig). The findings demonstrated that, compared to the Saline group, the MPTP group showed reduced expression of DLAT, DLD, PDHA1, FDX1, PDHB, GLRX5, and LIAS (*P *< 0.05), whereas HSPA1A expression was elevated (*P* < 0.05). In the MPTP-induced PD mouse model, lipoylation of DLAT and DLST was decreased compared to the Saline group (Fig 11J), and protein oligomers were more readily formed, although there is no significant difference, they show an upward trend (Fig 11K, 11L). While this trend aligns directionally with the proposed cuproptosis mechanism involving copper-induced DLAT oligomerization, the lack of statistical significance indicates this specific endpoint requires further validation under conditions of defined copper overload.

### Drug prediction and molecular docking

**Fig 12:**
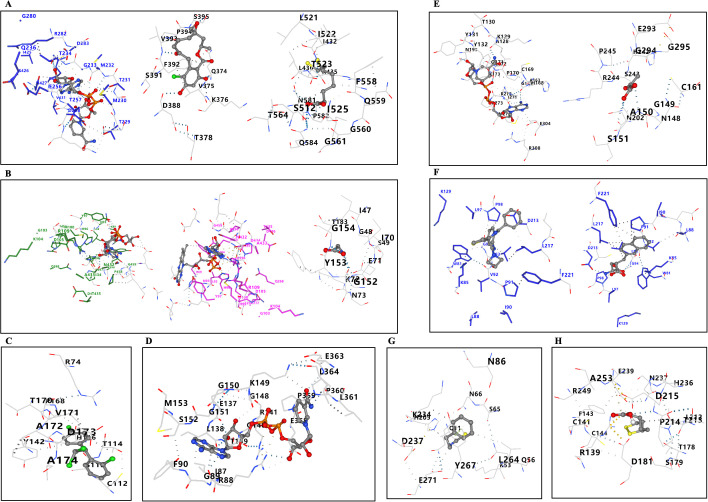
The results of mitochondrial dysfunction related proteins with potential therapeutic drug molecular docking. **(A)** DLAT docking results with NADH, Radicicol, and Dihydrolipoic Acid. **(B)** DLD docking results with NADH, Flavin adenine dinucleotide, and Glycine. **(C)** FDX1 docking results with Mitotane. **(D)** PDHA1 docking results with NADH. **(E)** PDHB docking results with NADH and Pyruvic acid. **(F)** GLRX5 docking results with DB08698 and DB08272. **(G)** HSPA1A docking results with Phenethyl Isothiocyanate. **(H)** LIAS docking results with Lipoic acid.

First, the three-dimensional structures of the proteins were retrieved from the PDB (S10 Fig), while the structures and pharmacological properties of the drugs were obtained from PubChem (S1 Table). We identified potential therapeutic drugs targeting mitochondrial dysfunction-related proteins that may be effective in treating PD. For instance, NADH, initially used as an adjunctive therapy for PD, has been shown in clinical trials to improve functional impairments in PD patients [36]. Radicipol exhibits antioxidant and neuroprotective properties, which can mitigate neuronal damage and inflammatory responses in PD [37]. Glycine has been found to effectively prevent nerve damage caused by stroke and reduce lipid peroxides in cerebrospinal fluid [38]. Subsequently, we conducted molecular docking analysis to evaluate the interactions between the proteins and the related drugs. The results, including vina scores, cavity volumes, and contact residues, are presented in Fig 12 and Table 3.

**Table 3:** The results of protein-drug molecular docking, including Vina score, Cavity volume, and Contact residues.

Protein	Drug	Vina score	Cavity volume (Å3)	Contact residues
**DLAT**	NADH	−6.9	–	Chain A: SER227 THR229 MET230 THR231 MET232 GLY233 THR234 GLU256 THR257 ASP258 LYS259 ARG282 ASP283 ASN427 ARG430 VAL431 GLN434
	Radicicol	−6.6	176	Chain A: GLN374 VAL375 LYS376 THR378 ASP388 SER391 PHE392 VAL393 PRO394 SER395
	Dihydrolipoic Acid	−4.3	2774	Chain A: ILE432 ARG435 LEU436 SER512 LEU521 ILE522 THR523 ILE525 PHE558 GLN559 GLY560 GLY561 THR564 ASN581 PRO582 GLN584
**DLD**	NADH	−7.4	–	Chain A: LYS89 LEU92 ASN93 HIS96 TYR97 MET100 ARG109 ARG234 TYR394 THR395 HIS396 GLU398 ASN432 ASP434 PRO458 GLY459 ALA460 GLY461 GLU462 LEU490
	Flavin adenine dinucleotide	−8.7	–	Chain A: ILE86 LYS89 ALA90 LEU92 ASN93 ASN94 HIS96 MET100 ARG109 GLN126 ALA130 ARG234 TYR394 THR395 HIS396 PRO397 GLU398 THR431 ASN432 ALA433 ASP434 PRO458 GLY459 GLU462 LEU490
	Glycine	−3.6	2461	Chain A: ILE47 GLY48 SER49 ILE70 GLU71 LYS72 ASN73 GLY152 TYR153 GLY154 THR183
**FDX1**	Mitotane	−5.8	311	Chain A: ARG74 CYS112 SER113 THR114 HIS116 TYR142 PRO168 THR170 VAL171 ALA172 ASP173 ALA174
**PDHA1**	NADH	−7.8	239	Chain A: ILE87 ARG88 GLY89 PHE90 GLU137 LEU138 THR139 GLY140 ARG141 GLY148 LYS149 GLY150 GLY151 SER152 MET153 GLU358 PRO359 PRO360 LEU361 GLU363 LEU364
**PDHB**	NADH	−8.0	606	Chain A: ALA128 LYS129 THR130 TYR131 TYR132 ALA163 GLY167 HIS168 CYS169 PRO170 GLY171 LEU172 LYS173 ASN195 ILE271 ARG272 PRO273 MET274 GLU304 ARG308
	Pyruvic acid	−4.1	768	Chain A: ASN148 GLY149 ALA150 SER151 CYS161 ASN202 HIS242 SER243 ARG244 PRO245 GLU293 GLY294 GLY295
**GLRX5**	DB08698	−7.0	–	Chain A: ALA40 PRO41 PRO44 PRO145 VAL148 PRO149 GLU152
	DB08272	−6.4	–	Chain A: TRP81 LYS85 LEU88 ASP89 ILE90 PRO91 VAL92 LEU93 GLY94 LEU97 PRO98 CYS210 ASP213 LEU217 PHE221
**HSPA1A**	Phenethyl Isothiocyanate	−5.6	5782	Chain A: GLN11 LYS53 GLN56 SER65 ASN66 ASN86 HIS205 LYS234 ASP237 LEU264 TYR267 GLU271
**LIAS**	Lipoic acid	−5.1	3943	Chain A: ARG139 CYS141 PHE143 CYS144 THR178 SER179 ASP181 LEU212 THR213 PRO214 ASP215 HIS236 ASN237 GLU239 ARG249 ALA253

## Conclusion

Copper, an essential metal for the human body, usually exists as Cu^2+^ in vivo. It is most concentrated in the brain, liver, and kidneys, with the brain being a key organ for its metabolism [39]. Copper takes part in numerous physiological processes, such as skin pigmentation, maintaining vascular integrity, myelin sheath formation, iron homeostasis, antioxidant defense, and neurotransmitter synthesis. Its significance in biological functions mainly comes from being a co – factor or structural part of various copper – containing proteins [40].

For instance, copper functions as a co-factor in Cu/Zn-SOD to scavenge ROS and aids electron transfer in the cytochrome C ETC between complex III and complex IV in the mitochondrial inner membrane. It’s also involved in neurotransmitter – related processes like synthesis (by dopamine β-hydroxylase) and metabolism (by diamine oxidase and monoamine oxidase), as well as in handling other metals (through metallothioneins, ceruloplasmin (CP), and hemoglobin) and extracellular matrix formation (by lysyl oxidase) [41].

Copper enters the bloodstream via the protein adenosine triphosphatase 7A (ATP7A), and ATP7B regulates its levels in tissues [42]. In recent years, radioactive and stable isotopes have helped us better understand copper metabolism [43]. Copper has two oxidation states, Cu^+^ and Cu^2+^, with Cu^+^ playing important roles in processes like iron homeostasis, oxygen metabolism, dopamine oxidation, and neurotransmitter synthesis [43]. CP, a copper – containing glycoprotein mainly synthesized in the liver, contains up to 95% of circulating copper [44]. Copper reacts with CP to convert Fe^2+^ to Fe^3+^, facilitating iron transport in plasma [45]. Moreover, copper can catalyze the Fenton reaction, promoting the formation of hydroxyl radicals: Cu^2+^+H_2_O_2_→Cu^+^+·O2H+H^+^, Cu^+^+H_2_O_2_→Cu^2+^+·OH + OH^-^.

Pretreatment with copper can increase the expression of copper – blue protein. It can prevent the loss of copper – blue protein oxidase activity caused by MPP+ and the subsequent rise in lipid peroxidation. Also, it slightly reduces iron content in the striatum and midbrain [46]. We think the increase in iron oxidase activity reduces iron content and prevents lipid peroxidation. Thus, copper – induced expression of copper – blue protein could be an experimental approach to counteract the harmful effects of iron deposits in PD. Copper can boost dopamine oxidation, forming potentially toxic substances [47]. PD is characterized by the selective loss of dopamine neurons in the SN and the presence of intracellular LBs, mainly made of α-syn fibrils [48,49]. Evidence shows excessive copper can cause neuronal cell death and α-syn aggregation [50].

Copper binding to α-syn is key for PD development, triggering several related effects. Firstly, it causes α-syn conformational changes, promoting fibrillation and aggregation [51,52]. Experiments indicate the copper-α-syn complex alters copper’s redox properties, increasing H_2_O_2_ production from ascorbic acid oxidation, which in turn oxidizes dopamine. The complex can also oxidize antioxidants like glutathione (GSH) [53]. A study by Davies and colleagues showed recombinant α-syn can bind both copper and iron, with copper altering iron binding kinetics, suggesting distinct metal binding sites. Also, α-syn has ferrireductase activity, linked to Fe(II) in dopamine synthesis and PD-related oxidative stress [54].

Injecting copper sulfate into rodents’ SN can be toxic to dopaminergic cells, reducing dopamine levels, increasing oxidative stress, causing apoptosis, and decreasing TH immunoreactivity [55]. Several groups found higher copper concentrations in PD patients’ cerebrospinal fluid than in controls [56,57]. Free copper in cerebrospinal fluid may relate to clinical variables and could be a disease biomarker [58].

The downregulation of DEC-MDRGs (DLAT, DLD, PDHA1, etc.) and mitochondrial structural damage are robust findings supporting mitochondrial dysfunction. The role of copper could involve other pathways (e.g., oxidative stress via ROS generation, disruption of iron-sulfur clusters, or indirect effects on expression) alongside or instead of direct DLAT oligomerization in this model. Our data confirm significant mitochondrial damage and downregulation of key metabolic enzymes (DLAT, DLD, PDHA1, etc.) in the MPTP model. While direct copper-mediated DLAT oligomerization was not significantly increased, copper dyshomeostasis likely contributes to pathology through elevated ROS, impaired antioxidant defense (reduced GLRX5, LIAS), and disruption of TCA cycle function. Similar results were found in mice serum. We also found potential PD drugs like NADH, Radicipol, and Glycine, which act on oxidative stress, inflammation, and mitochondrial function.

DLAT and DLD are in mitochondrial dihydrolipoamide acetyltransferase, involved in oxidative phosphorylation for energy production. FDX1 is an iron-sulfur cluster protein in redox reactions during electron transfer. PDHA1 and PDHB are in the pyruvate dehydrogenase complex, important for glucose metabolism. GLRX5 regulates cellular redox balance. HSPA1A is a heat shock protein, involved in stress response. LIAS is key for mitochondrial fatty acid synthesis, maintaining mitochondrial function. In short, these genes’ relation to copper-induced cell death is in their connection with copper ion regulation, mitochondrial dysfunction, and PD development, and could be PD treatment biomarkers and guide therapy research.

Copper is a catalytic co-factor in energy metabolism, mitochondrial respiration, and antioxidant defense [59]. Usually, intracellular Cu concentration is low. But when it accumulates, excessive Cu binds to mitochondrial proteins, causing protein toxicity and oxidative stress-induced cell death. Copper-dependent cell death happens when copper binds directly to lipoylation components of the TCA cycle [10], leading to lipoylated protein aggregation and loss of iron-sulfur cluster proteins, and finally cell death. FDX1 regulates protein lipoylation upstream. Copper binds and oligomerizes DLAT, and without FDX1, DLAT and DLST don’t bind to copper, showing the lipoyl moiety is vital for copper binding [10].

Protein lipoylation is crucial in many biological processes. In signaling pathways, it helps key proteins localize to the cell membrane and activate downstream signals. In immune responses, lipoylated proteins can trigger immune reactions. Abnormal lipoylated protein levels are seen in neurodegenerative diseases like Alzheimer’s and Huntington’s, suggesting a role in neuroprotection and anti-aging [60]. Current data suggest both high and low copper levels are harmful, and proper homeostasis control is essential [61–63]. Abnormal copper homeostasis, due to genetic mutation, aging, or environment, leads to pathologies like cancer, inflammation, and neurodegeneration [64,65].

In addition to the predicted drugs such as NADH and Radicipol, this study provides the following new directions for PD prevention and treatment. In terms of early diagnosis, the identified DEC-MDRGs (such as DLAT, FDX1, and PDHA1), which are abnormally expressed in PBMCs of PD patients, can serve as non-invasive diagnostic biomarkers, particularly suitable for screening high-risk populations. For copper homeostasis regulation, interventions can be implemented through two approaches: dietary adjustments and gene therapy. Dietary interventions may involve maintaining brain copper balance by regulating copper intake or using copper chelators (such as penicillamine) to prevent mitochondrial damage. Gene therapy could target key genes (such as the copper transporter SLC31A1 or miR-4632/miR-637) to restore copper metabolic homeostasis. Regarding mitochondrial function enhancement strategies, exercise intervention has been demonstrated to improve mitochondrial biogenesis in PD patients [66,67]. Metabolic reprogramming via activating the TCA cycle (e.g., supplementing α-lipoic acid) can effectively alleviate the energy crisis induced by cuproptosis.

The process by which abnormal copper drives PD through mitochondrial pathways involves multiple key mechanisms. In terms of direct toxicity, excessive copper ions (Cu^+^) target and bind to lipoylated TCA cycle proteins such as DLAT and DLST, inducing their oligomerization. This not only blocks energy metabolism but also leads to the loss of iron-sulfur cluster proteins [10]. At the level of oxidative stress amplification, copper generates ROS through the Fenton reaction, directly damaging mitochondrial DNA, membrane phospholipids, and respiratory chain complexes. Meanwhile, dysfunction of key antioxidant enzymes such as SOD and GLRX5 further exacerbates oxidative damage. Energy crisis and neuronal death manifest as reduced ATP synthesis due to interrupted TCA cycle (downregulation of DLAT/PDHA1) and ETC dysfunction (loss of FDX1), as well as mitochondrial membrane potential collapse triggering apoptotic pathways that selectively kill high-energy-consuming dopaminergic neurons. This study systematically elucidated the above mechanisms through multi-omics analysis and experimental validation (electron microscopy showing mitochondrial cristae fragmentation, western blotting confirming metabolic enzyme downregulation), demonstrating that targeting the mitochondrial-copper axis represents a critical therapeutic entry point for PD. For the first time, this research integrates the perspectives of cuproptosis and mitochondrial dysfunction to provide novel biomarkers (DEC-MDRGs) and multi-dimensional intervention strategies (pharmacological, genetic, and metabolic regulation) for PD. Future work will deepen the study of sex differences and actively promote the clinical translation of these research findings.

This knowledge offers a new field for neurodegenerative disease therapies via copper supplementation or removal. However, more research is needed, like regulating copper homeostasis to prevent neurodegeneration, deciding when to use copper chelation or supplementation, which can be answered by studying copper-dependent neurotoxic mechanisms and modulating factors. Serum metabolite screening can detect PD patients early for early intervention, support personalized therapy, and reveal metabolic pathways and molecular mechanisms, helping understand PD causes and develop new treatments.

## Supporting information

S1 Fig Box plots of immune cells with no significant differences, including the (A) GSE22491 and (B) GSE99039 datasets. (TIF)

S2 Fig The top 50 hub genes among the 9 modules. The lines in the network plot represent the degree of connectivity between genes, with thicker lines indicating stronger associations.
(TIF)

S3 Fig Protein-protein network analysis of differentially expressed genes. (TIF)

S4 Fig Enrichment analysis of biological functions and pathways of DECRGs. (A) Biofunctional enrichment analysis, including biological processes (BPs), cellular components (CCs), and molecular functions (MFs). (B) The enriched item in the KEGG. The size of circles represents the number of enriched genes.
(TIF)

S5 Fig Differential expression analysis of DEC-MDRGs. (A) The expression and correlation analysis of 12 DECRGs. (B) Venn diagram showing the overlap between hub genes from mitochondrial dysfunction-related genes (MDRGs) and cuproptosis-related genes (CRGs). (C) Differential expression analysis of DEC-MDRGs in GSE22491 dataset. (D) Fold change analysis of DEC-MDRGs in GSE49036 dataset. (E) Fold change analysis of HSPA1A in GSE99039, GSE20141, GSE20163, and GSE7621 datasets. Compared with healthy controls, *P < 0.05, **P < 0.01, ***P < 0.001, ****P < 0.0001.
(TIF)

S6 Fig Pathological analysis of DEC-MDRGs, including DLAT, DLD, FDX1, PDHA1, PDHB, GLRX5, HSPA1A, and LIAS 。Widely distributed in epithelial cells, glial cells, neuronal cells, and neutrophils.
(TIF)

S7 Fig The average expression level of proteins in different brain regions at different developmental period, including (A) DLAT, (B) DLD, (C) GLRX5, and (D) HSPA1A. (TIF)

S8 Fig Different cell types of protein expression in the brain during infancy and adulthood, including (A) DLAT, (B) DLD, (C) FDX1, (D) PDHA1, (E) PDHB, (F) GLRX5, (G) HSPA1A, and (H) LIAS. (TIF)

S9 Fig Immunohistochemical analysis of expression of mitochondrial dysfunction-related proteins in the MPTP-induced mice model in vivo of Parkinson’s disease. (A) Immunohistochemical and statistical analysis of DLAT protein. (B) Immunohistochemical and statistical analysis of DLD protein. (C) Immunohistochemical and statistical analysis of PDHA1 protein. (D) Immunohistochemical and statistical analysis of FDX1 protein. (E) Immunohistochemical and statistical analysis of LIAS protein. Compared with the Saline group, *P < 0.05, **P < 0.01, ***P < 0.001.
(TIF)

S10 Fig The structure of mitochondrial dysfunction related proteins. (TIF)

S1 File Original Western Blots. (DOCX)

## Abbreviation:

PD: Parkinson’s disease
SN: Substantia nigra
LBs: Lewy bodies
α-syn: α-synuclein
ROS: Reactive oxygen species
ATP: Adenosine triphosphate
SOD: Superoxide dismutase
ETC: Electron transport chain
CNS: Central nervous system
DEGs: Differentially expressed genes
PBMCs: Peripheral blood mononuclear cells
CRGs: Cuproptosis-related genes
MDRGs: Mitochondrial dysfunction-related genes
DEC-MDRGs: Differentially expressed cuproptosis-mitochondrial dysfunction-related genes
ROC: Receiver operating characteristic
miRNAs: microRNAs
SNpc: Substantia nigra pars compacta
UMAP: Uniform manifold approximation and projection
GSEA: Gene Set Enrichment Analysis
WGCNA: Weighted correlation network analysis
PPI: Protein-protein interaction
KEGG: Kyoto Encyclopedia of Genes and Genomes pathways
GO: Gene Ontology
DECRGs: Differentially expressed cuproptosis-related genes
ROC: Receiver operating characteristic
AUC: Area under the curve
TEM: Transmission electron microscopy
BPs: Biological processes
CCs: cellular components
MFs: Molecular functions
TCA: Tricarboxylic acid
GSH: Glutathione
