# Supplementary figures and images for: Cuproptosis-related genes associated with mitochondrial dysfunction in Parkinson’s disease
Source: PLoS One. 2025 Jul 17;20(7):e0327550. doi: 10.1371/journal.pone.0327550 (PMC12270184; doi:10.1371/journal.pone.0327550)

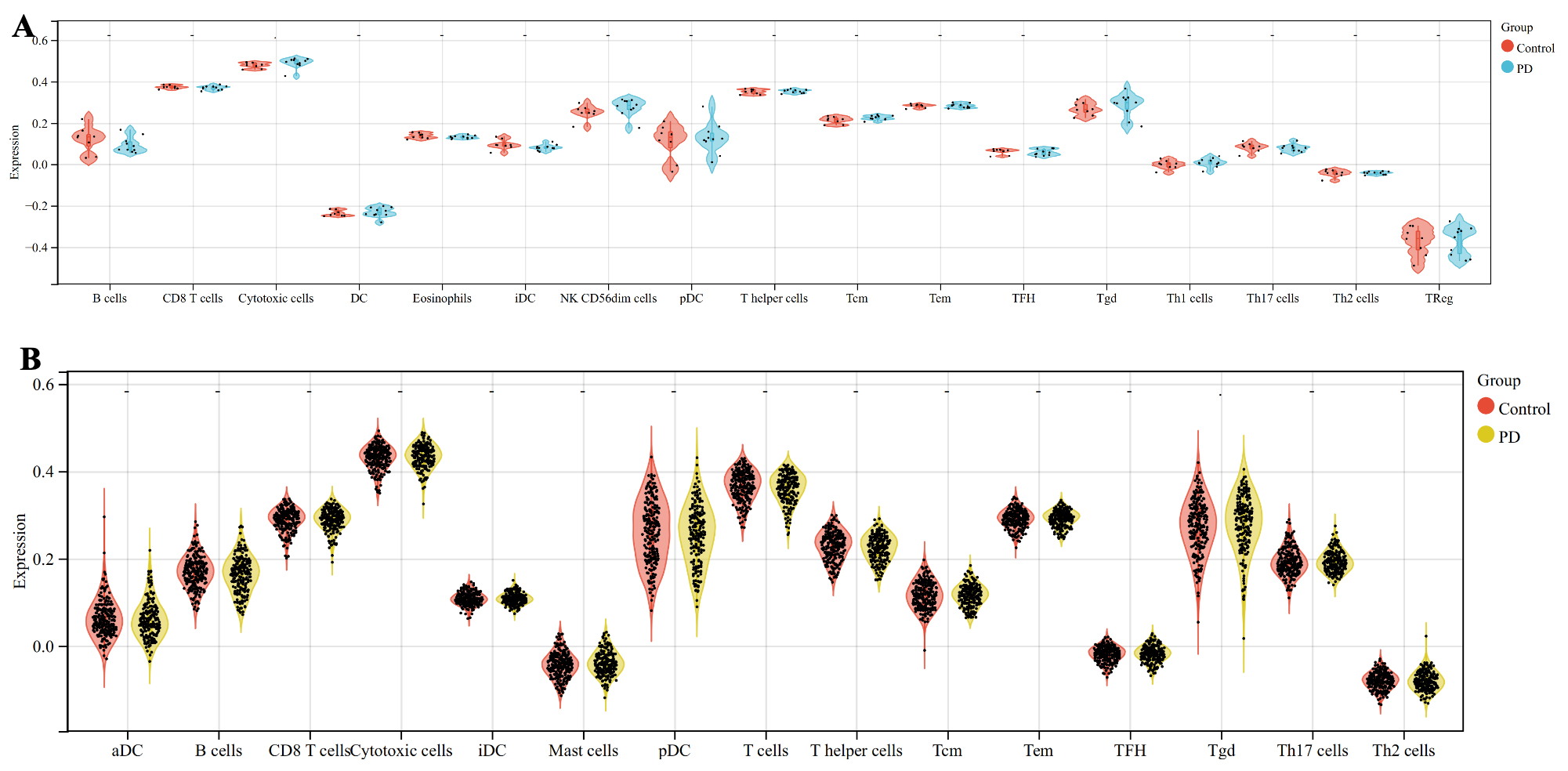

Supplement: S1 Fig — (TIF) [file pone.0327550.s001.tif]

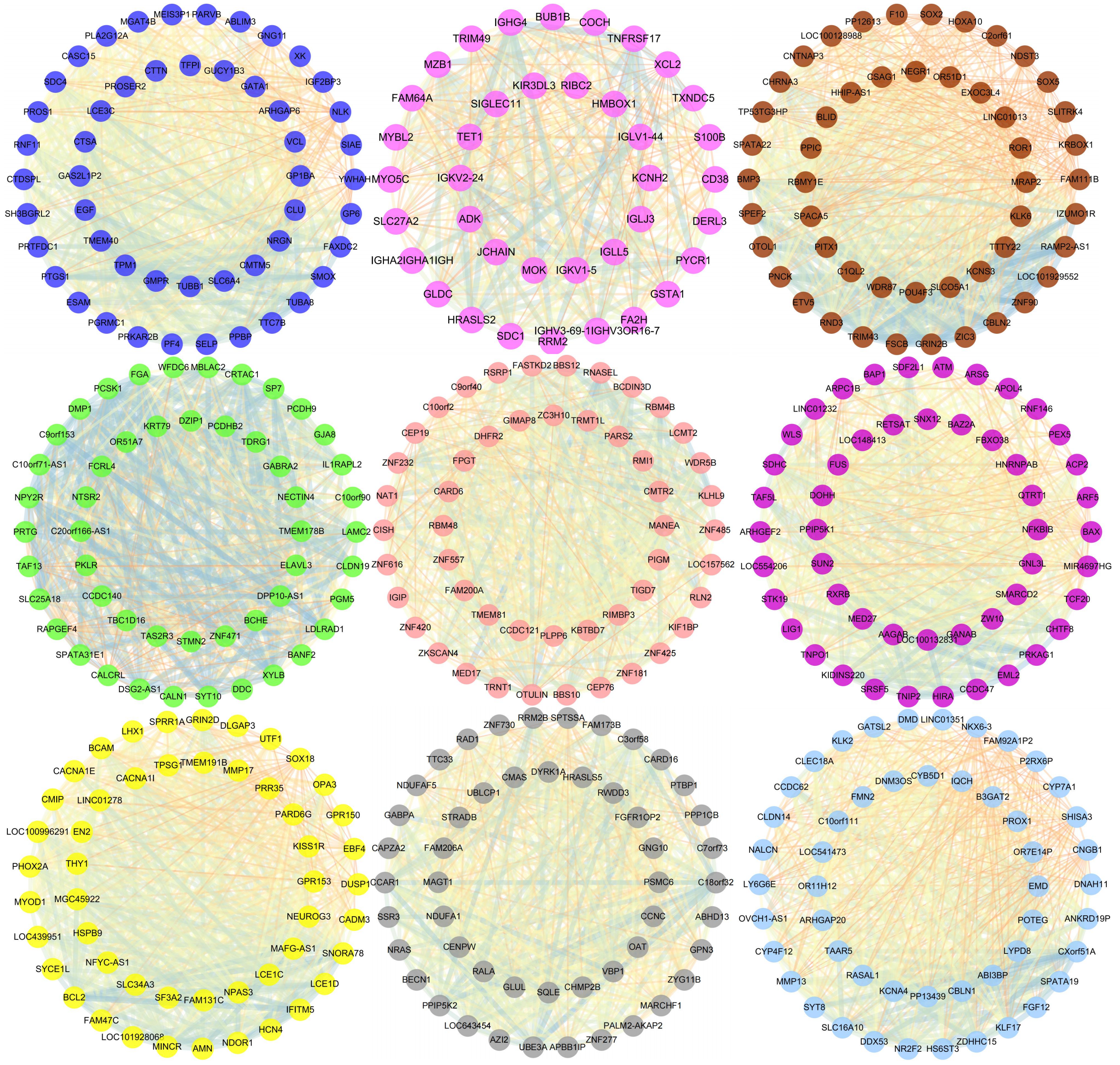

Supplement: S2 Fig — The lines in the network plot represent the degree of connectivity between genes, with thicker lines indicating stronger associations. (TIF) [file pone.0327550.s002.tif]

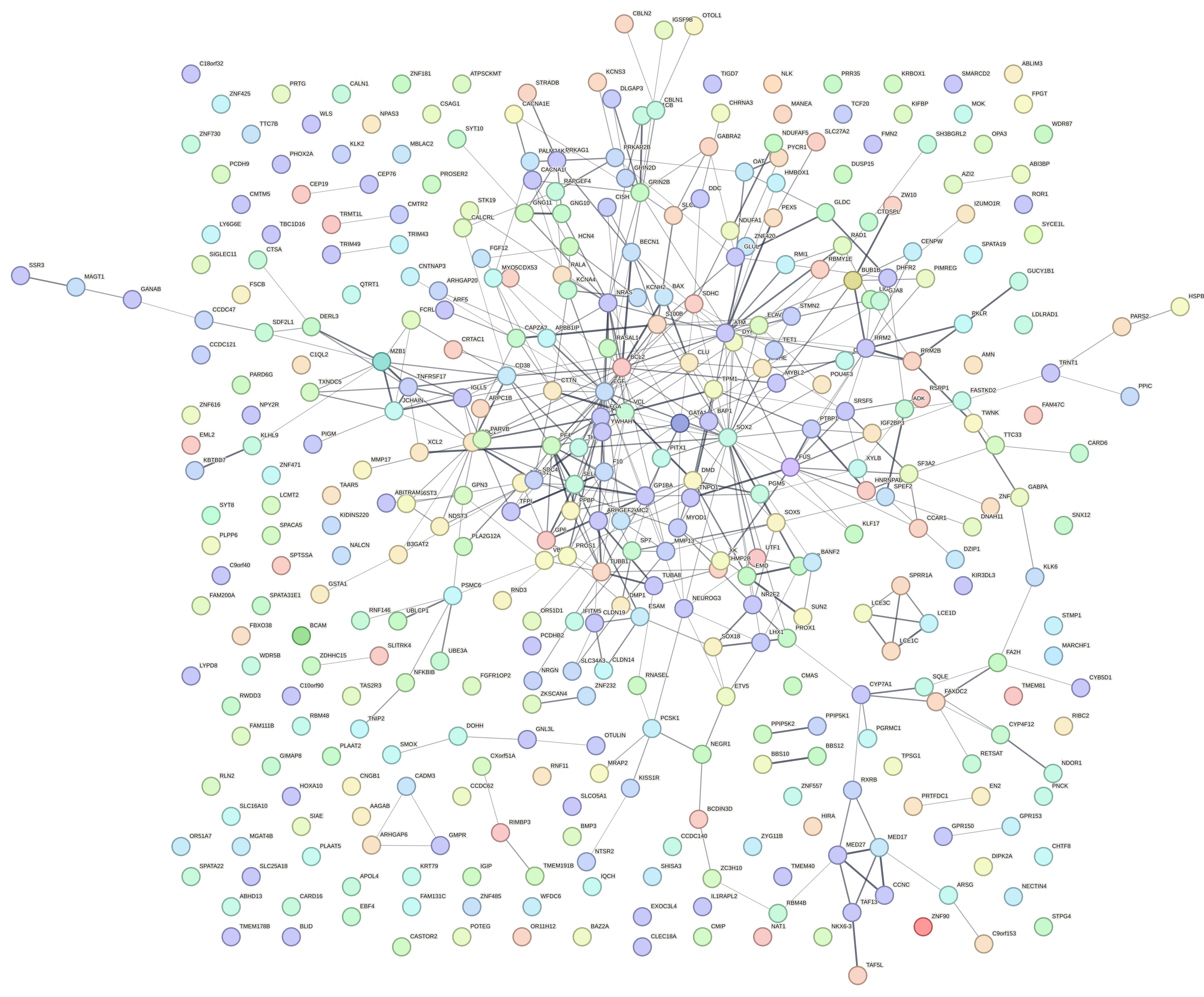

Supplement: S3 Fig — (TIF) [file pone.0327550.s003.tif]

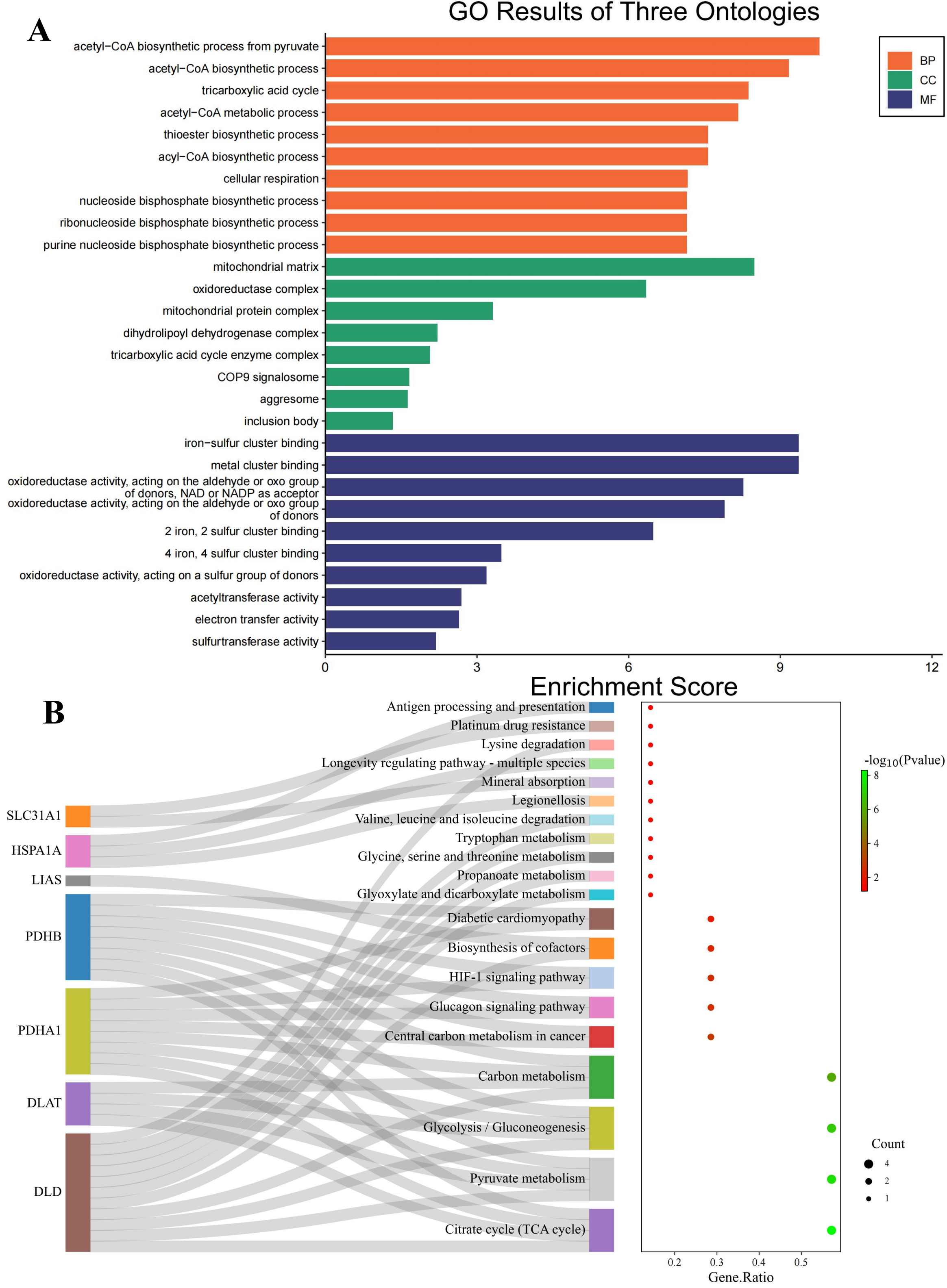

Supplement: S4 Fig — (A) Biofunctional enrichment analysis, including biological processes (BPs), cellular components (CCs), and molecular functions (MFs). (B) The enriched item in the KEGG. The size of circles represents the number of enriched genes. (TIF) [file pone.0327550.s004.tif]

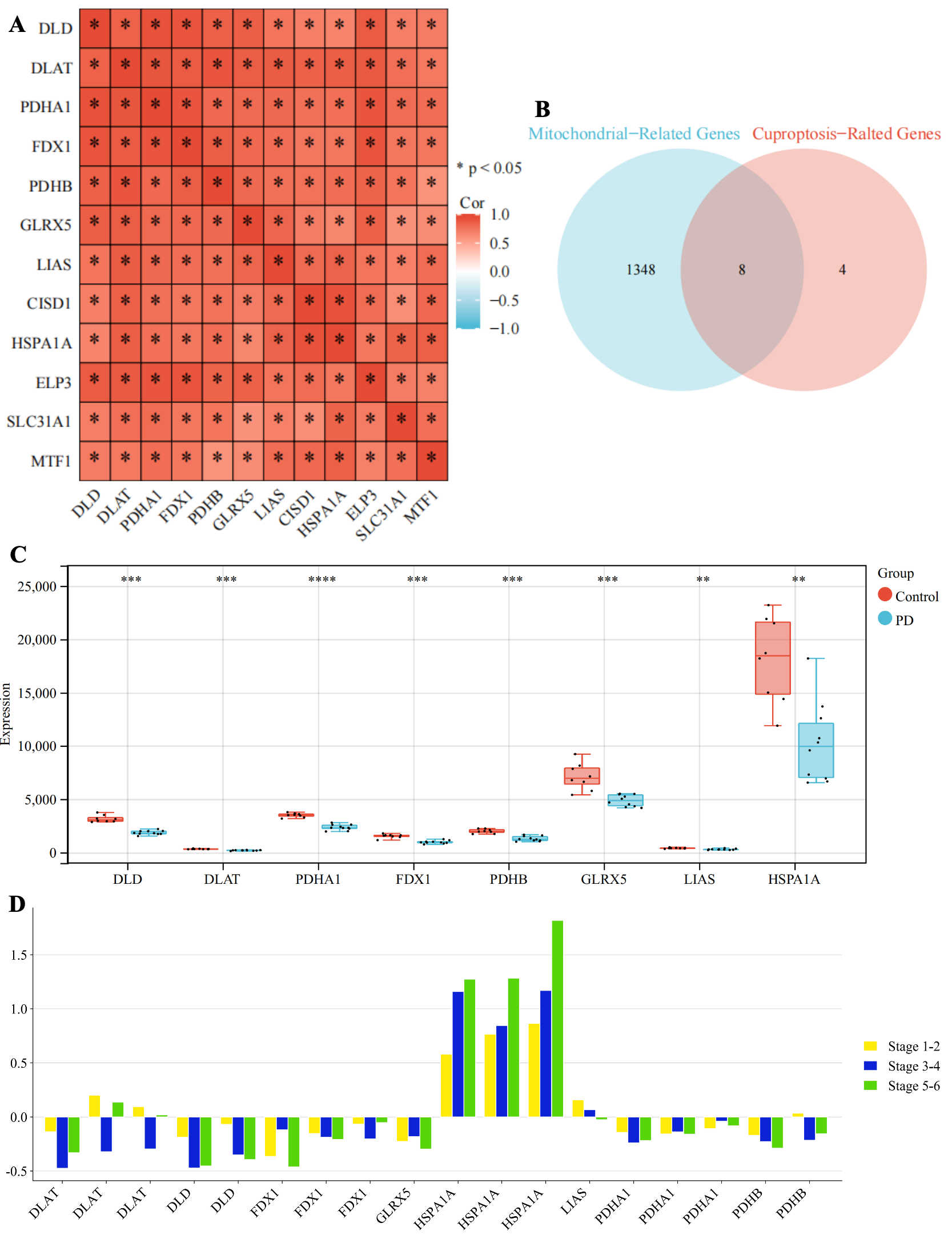

Supplement: S5 Fig — (A) The expression and correlation analysis of 12 DECRGs. (B) Venn diagram showing the overlap between hub genes from mitochondrial dysfunction-related genes (MDRGs) and cuproptosis-related genes (CRGs). (C) Differential expression analysis of DEC-MDRGs in GSE22491 dataset. (D) Fold change analysis of DEC-MDRGs in GSE49036 dataset. (E) Fold change analysis of HSPA1A in GSE99039, GSE20141, GSE20163, and GSE7621 datasets. Compared with healthy controls, *P < 0.05, **P < 0.01, ***P < 0.001, ****P < 0.0001. (TIF) [file pone.0327550.s005.tif]

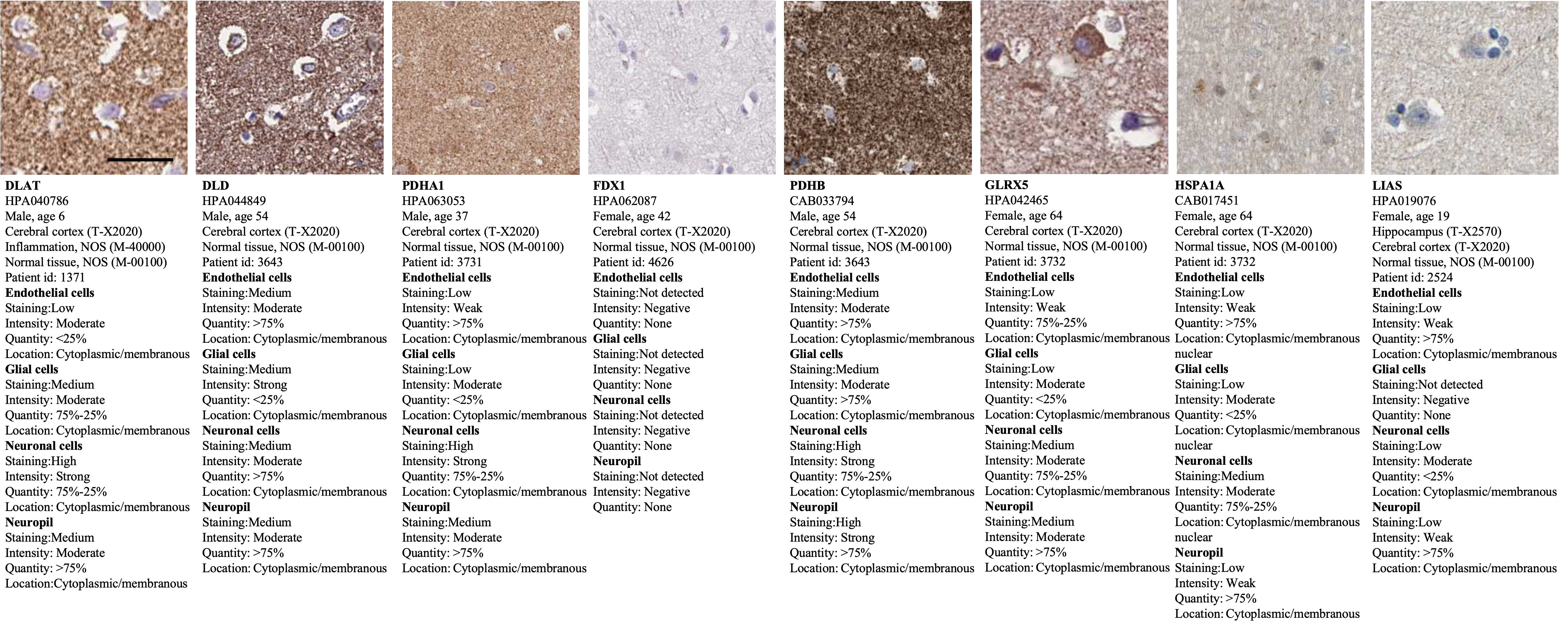

Supplement: S6 Fig — 。Widely distributed in epithelial cells, glial cells, neuronal cells, and neutrophils. (TIF) [file pone.0327550.s006.tif]

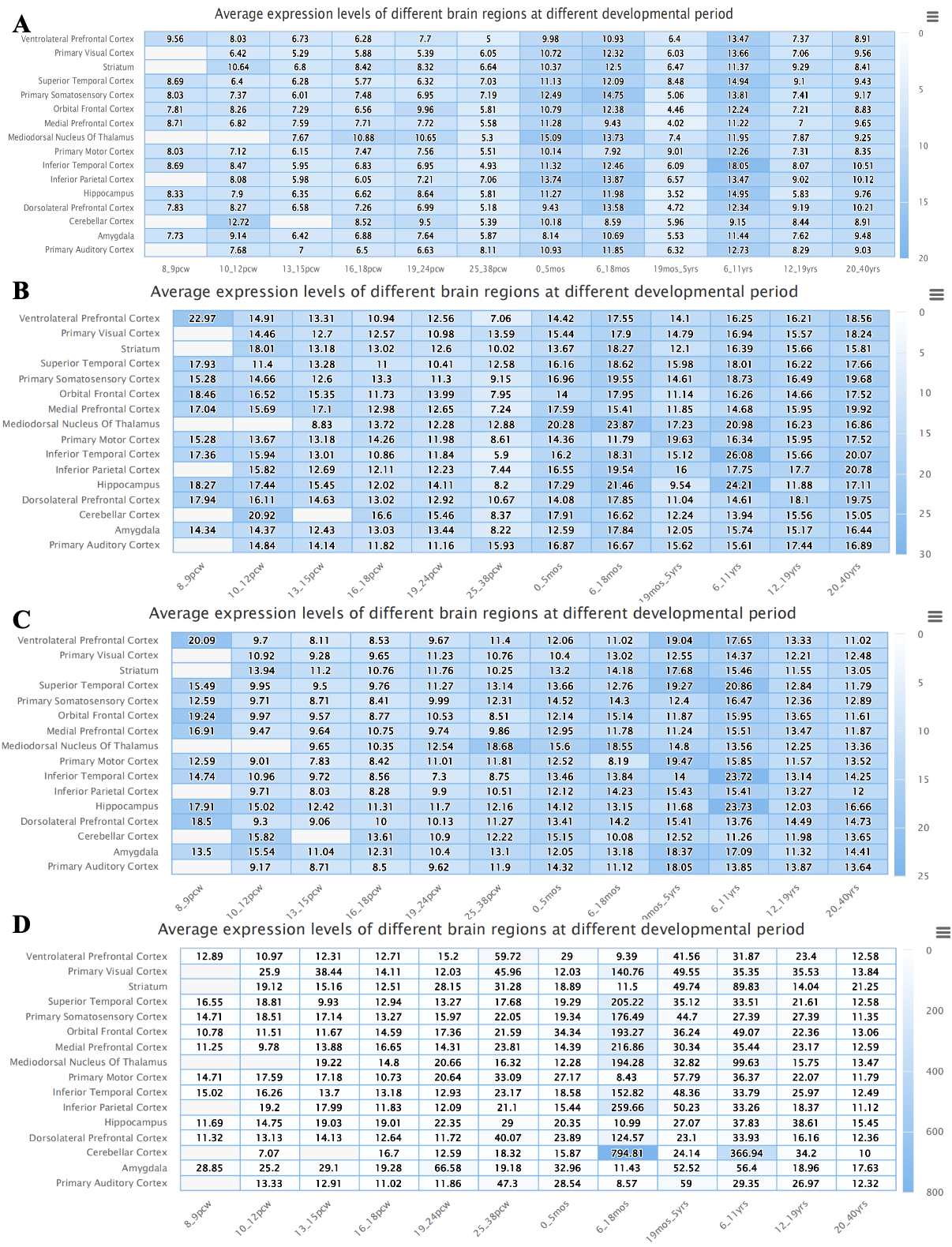

Supplement: S7 Fig — (TIF) [file pone.0327550.s007.tif]

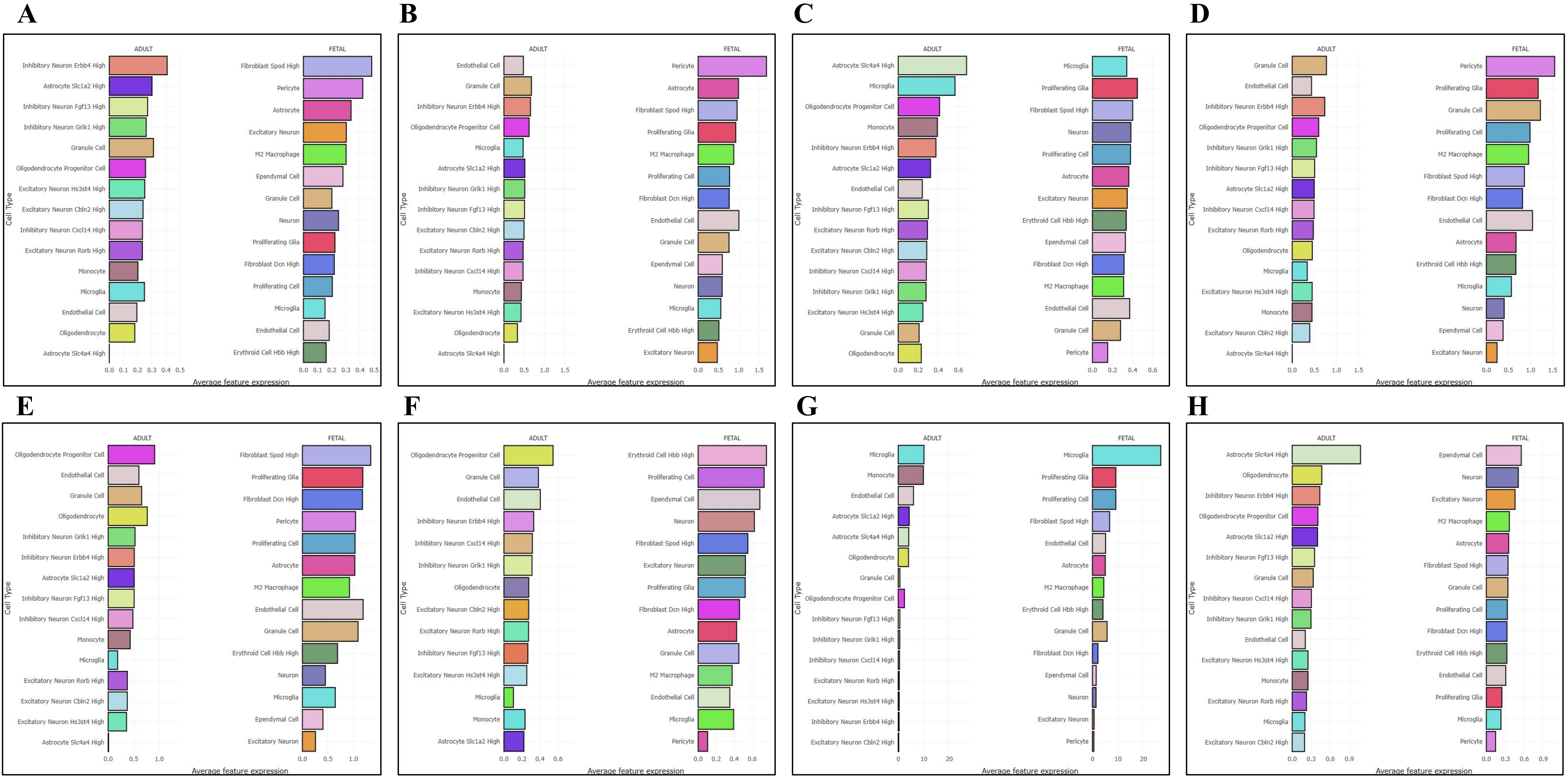

Supplement: S8 Fig — (TIF) [file pone.0327550.s008.tif]

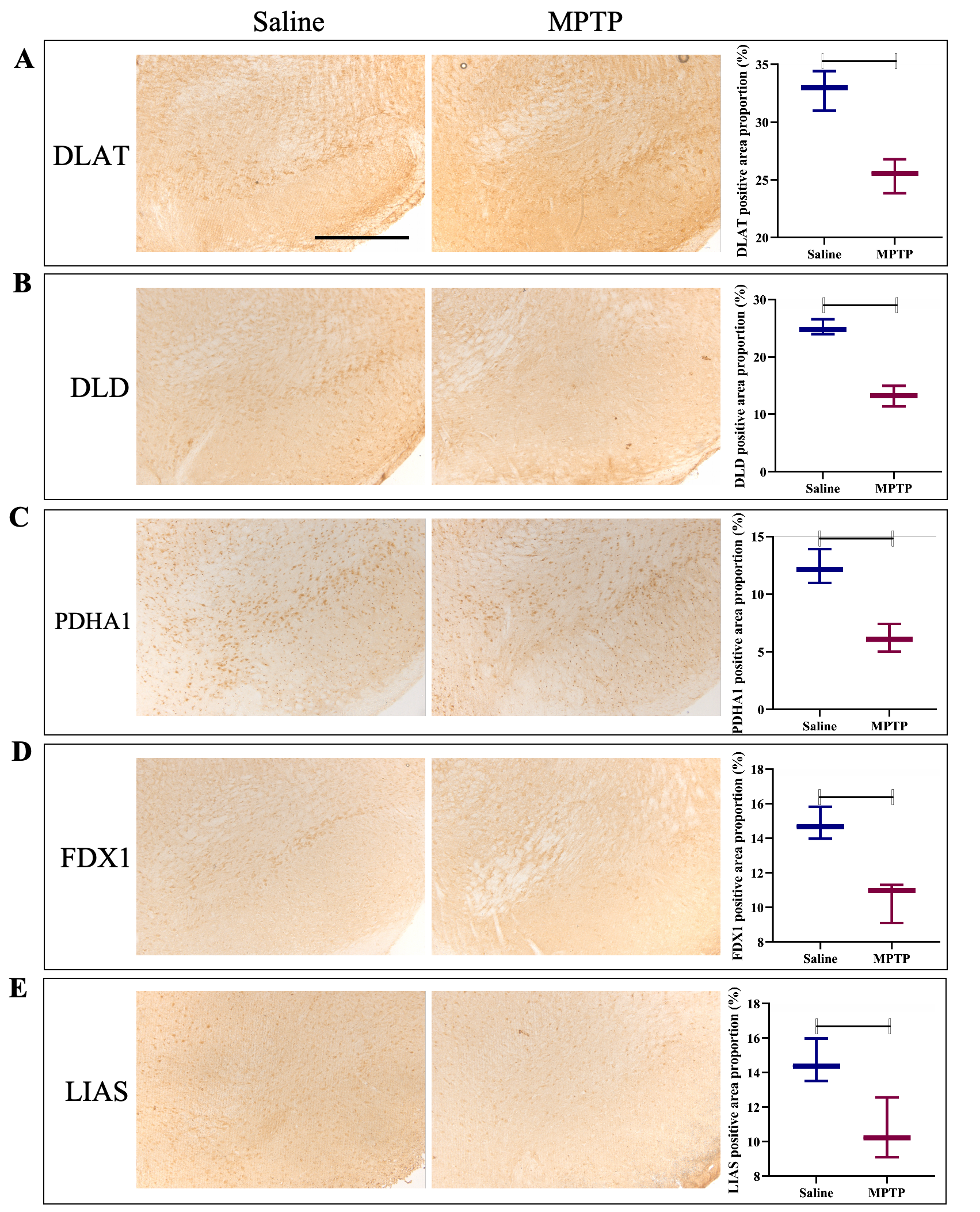

Supplement: S9 Fig — (A) Immunohistochemical and statistical analysis of DLAT protein. (B) Immunohistochemical and statistical analysis of DLD protein. (C) Immunohistochemical and statistical analysis of PDHA1 protein. (D) Immunohistochemical and statistical analysis of FDX1 protein. (E) Immunohistochemical and statistical analysis of LIAS protein. Compared with the Saline group, *P < 0.05, **P < 0.01, ***P < 0.001. (TIF) [file pone.0327550.s009.tif]

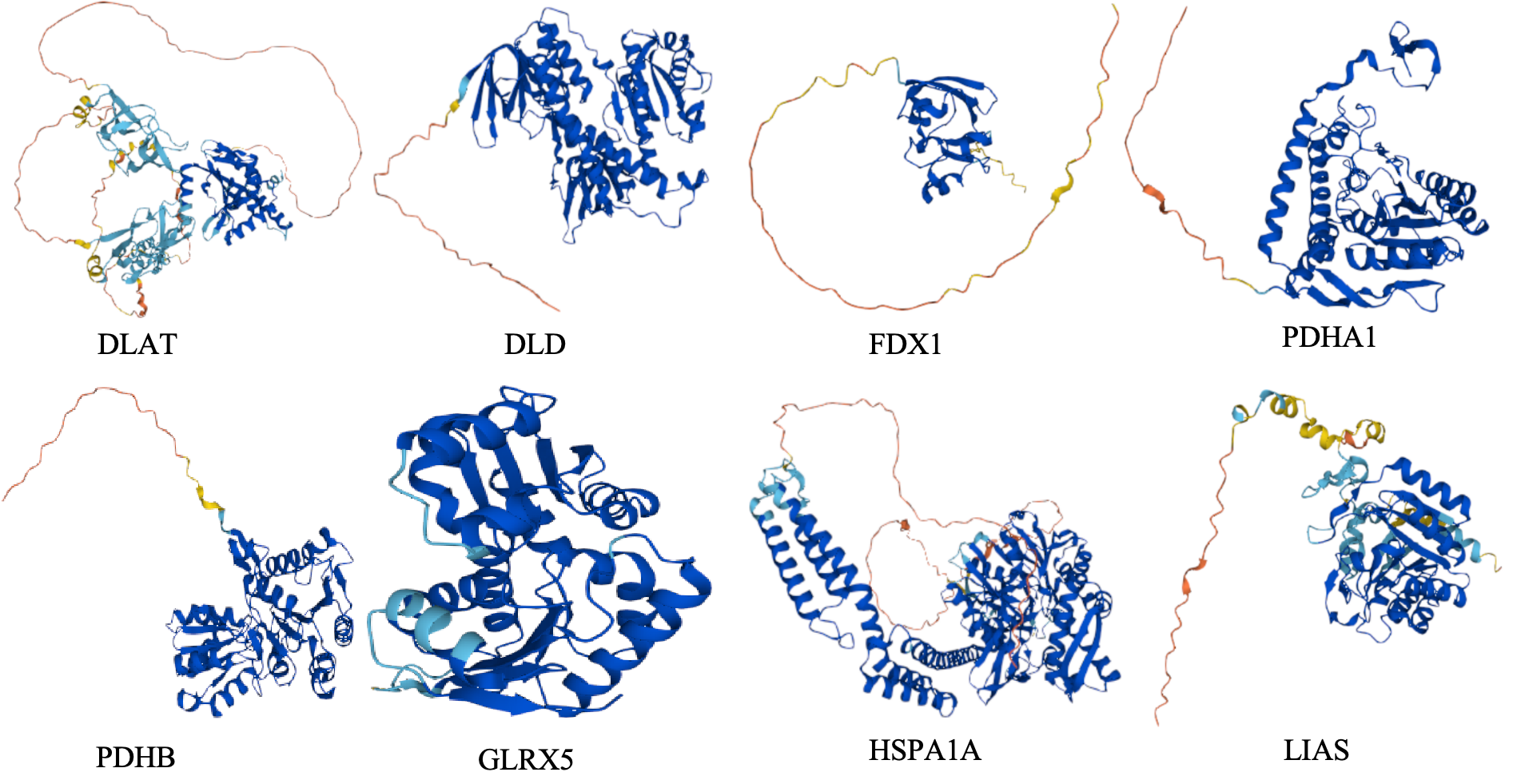

Supplement: S10 Fig — (TIF) [file pone.0327550.s010.tif]

| DLAT | 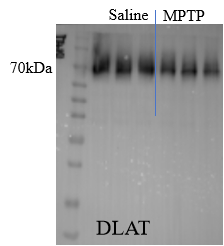 | 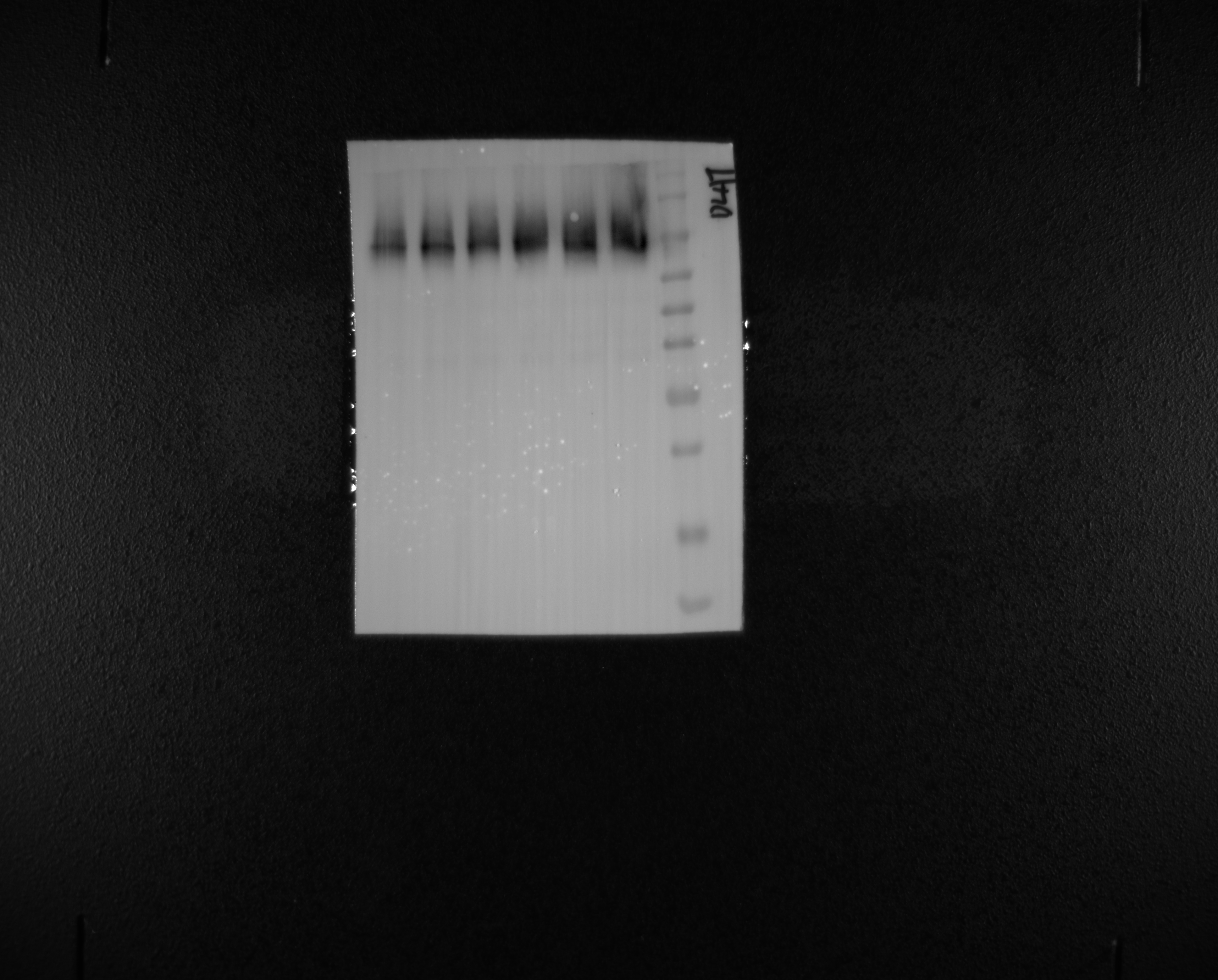 |
| --- | --- | --- |
| DLD | 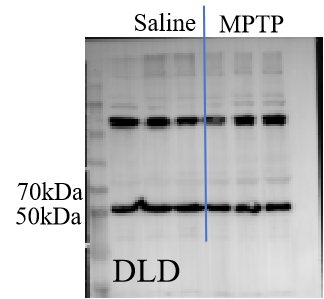 | 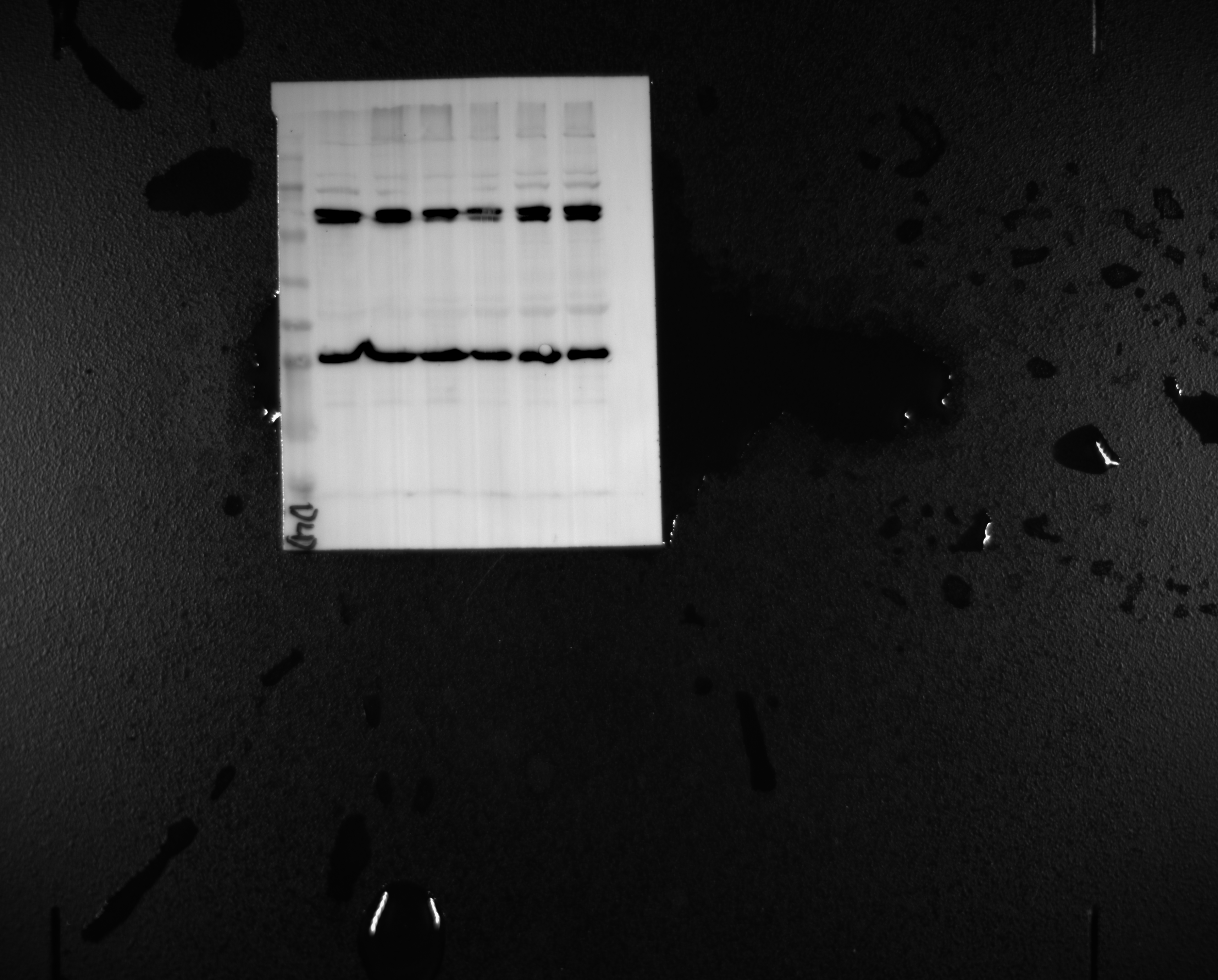 |
| PDHA1 | 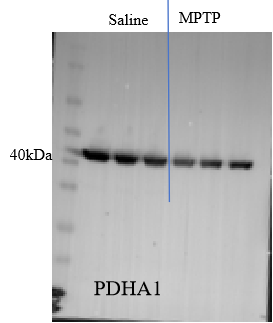 | 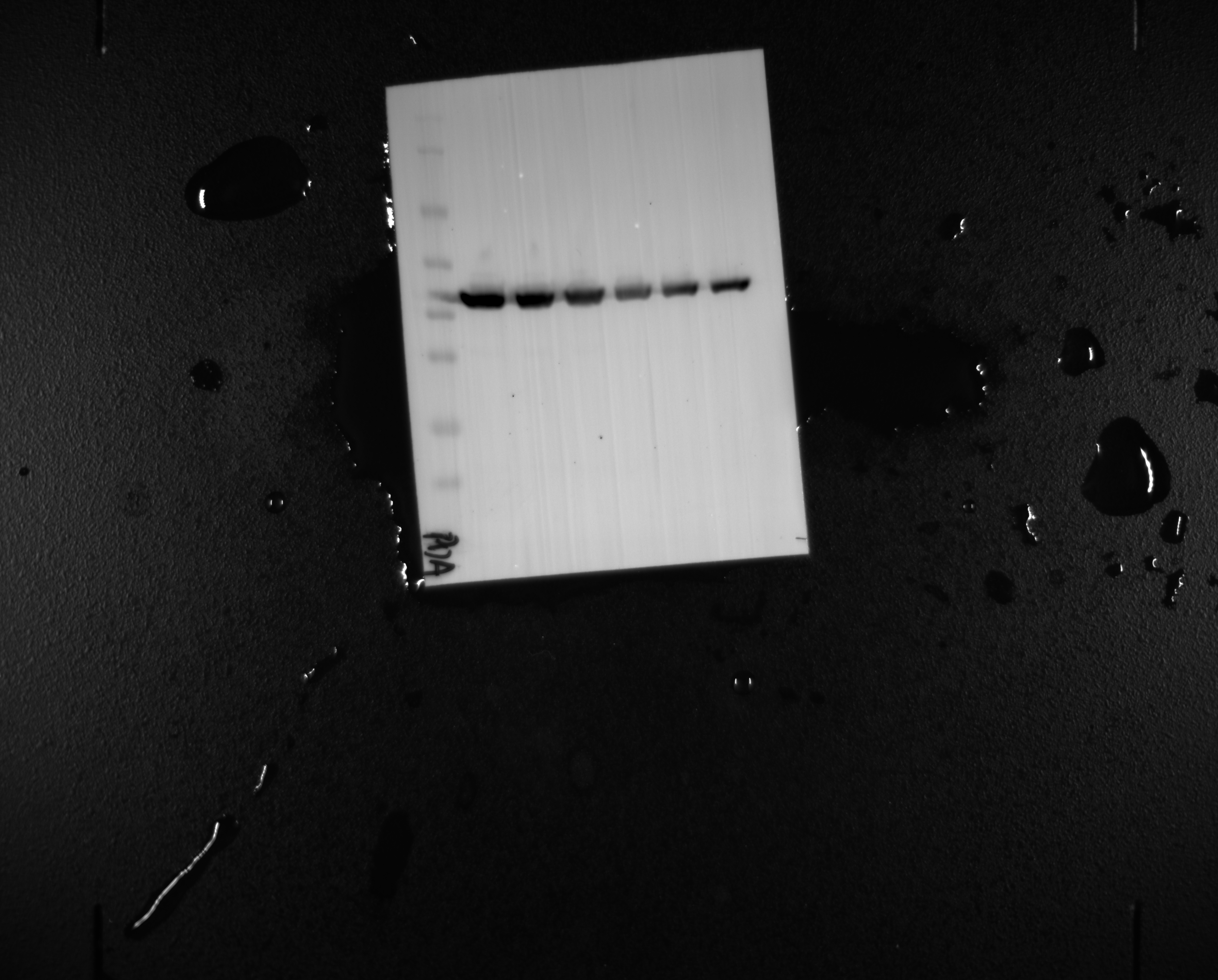 |
| FDX1 | 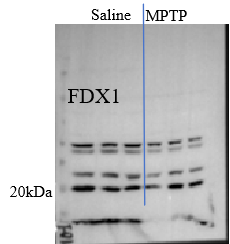 | 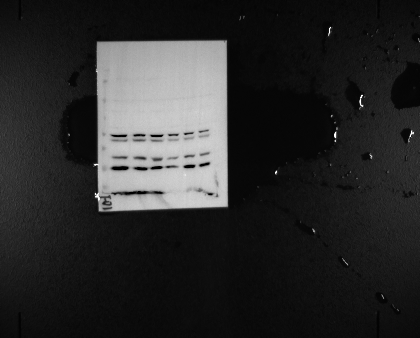 |
| PDHB | 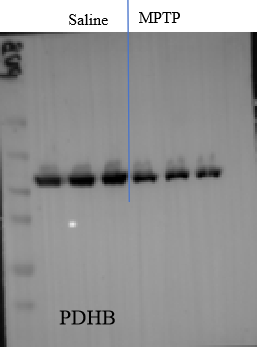 | 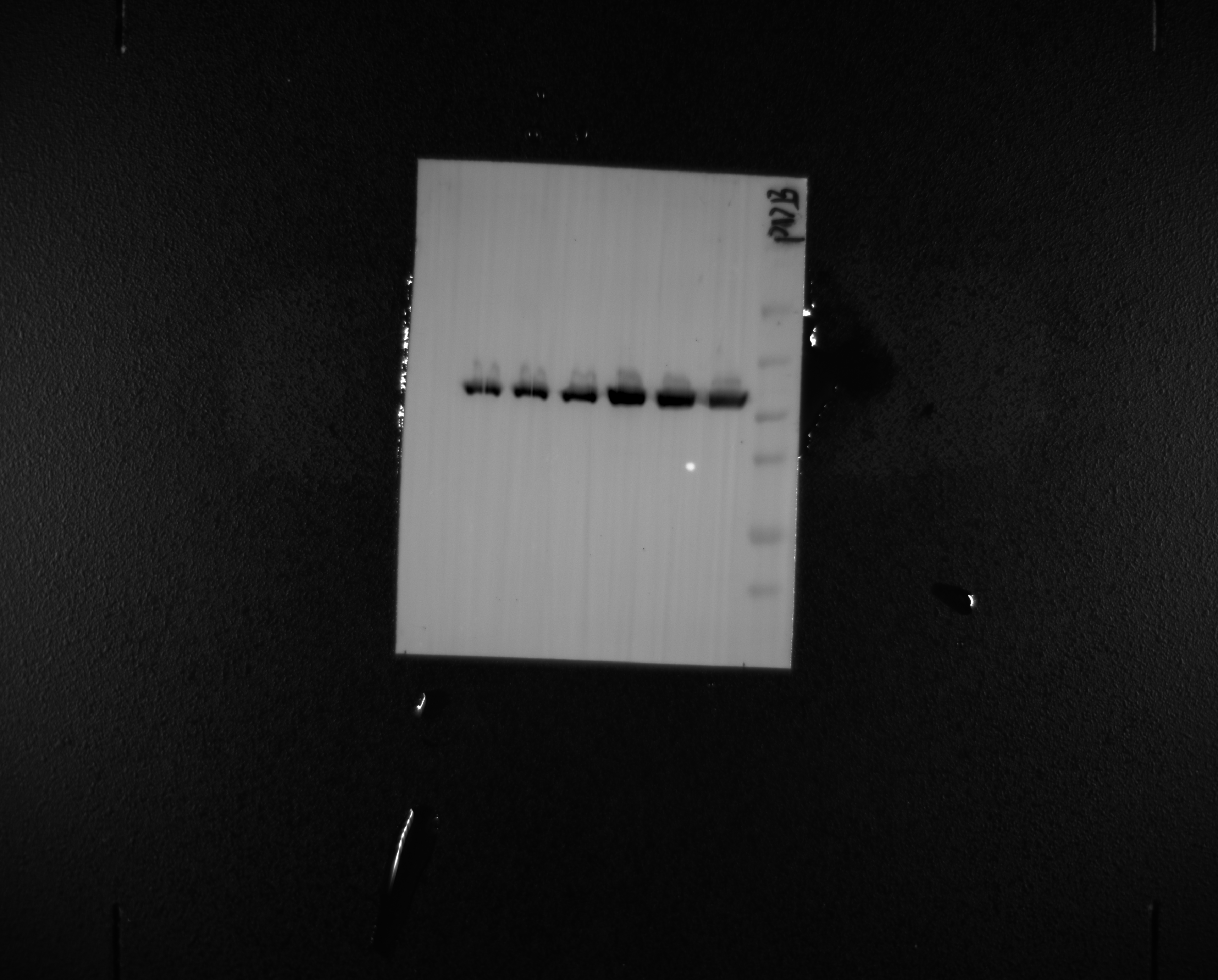 |
| GLRX5 | 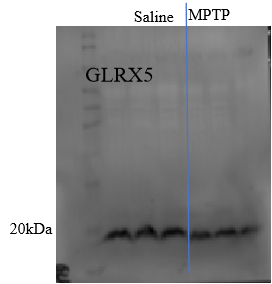 | 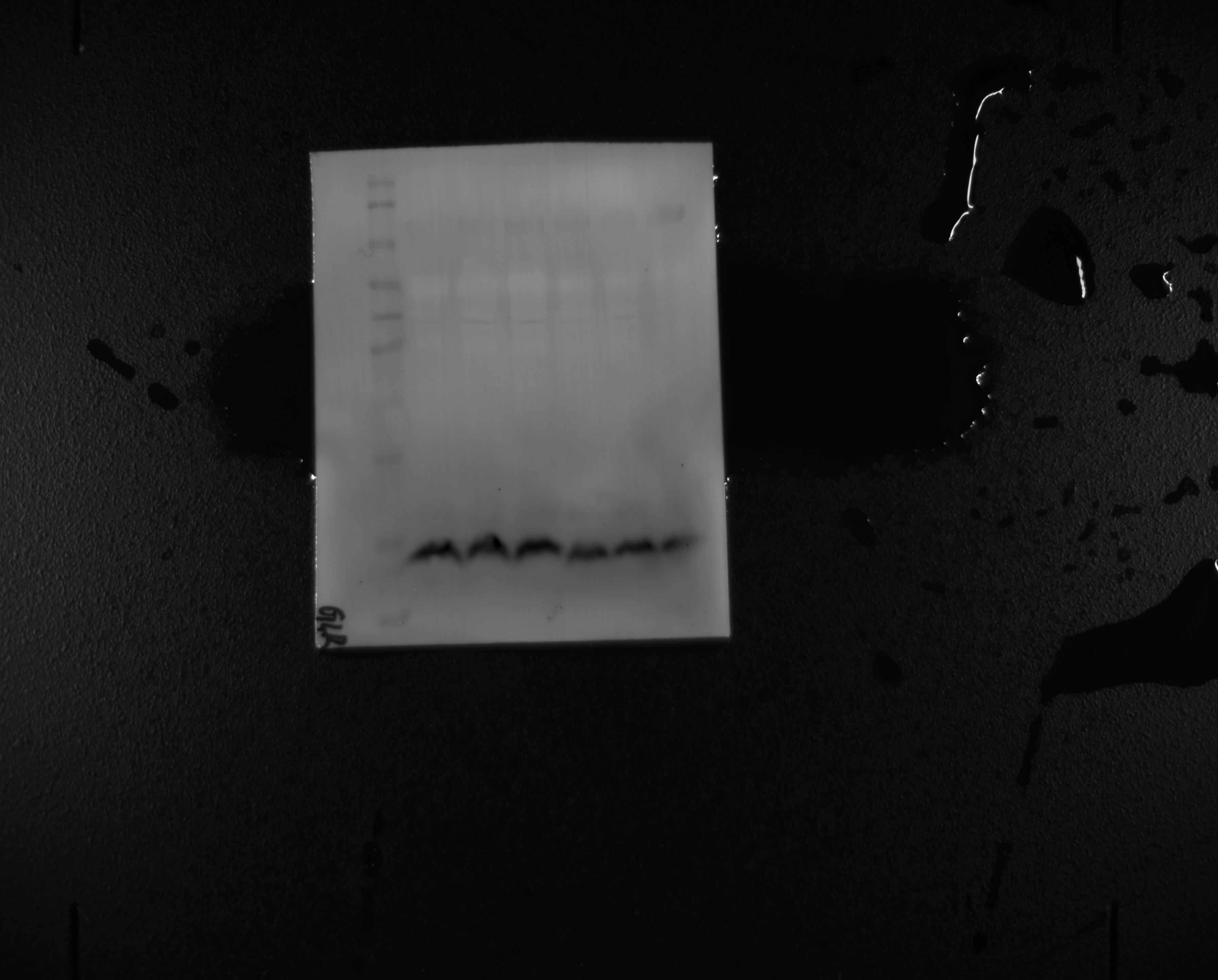 |
| HSPA1A | 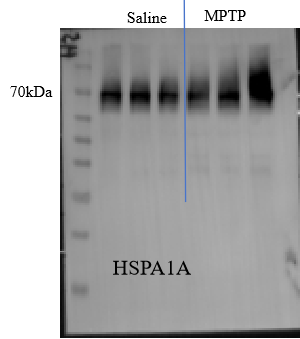 | 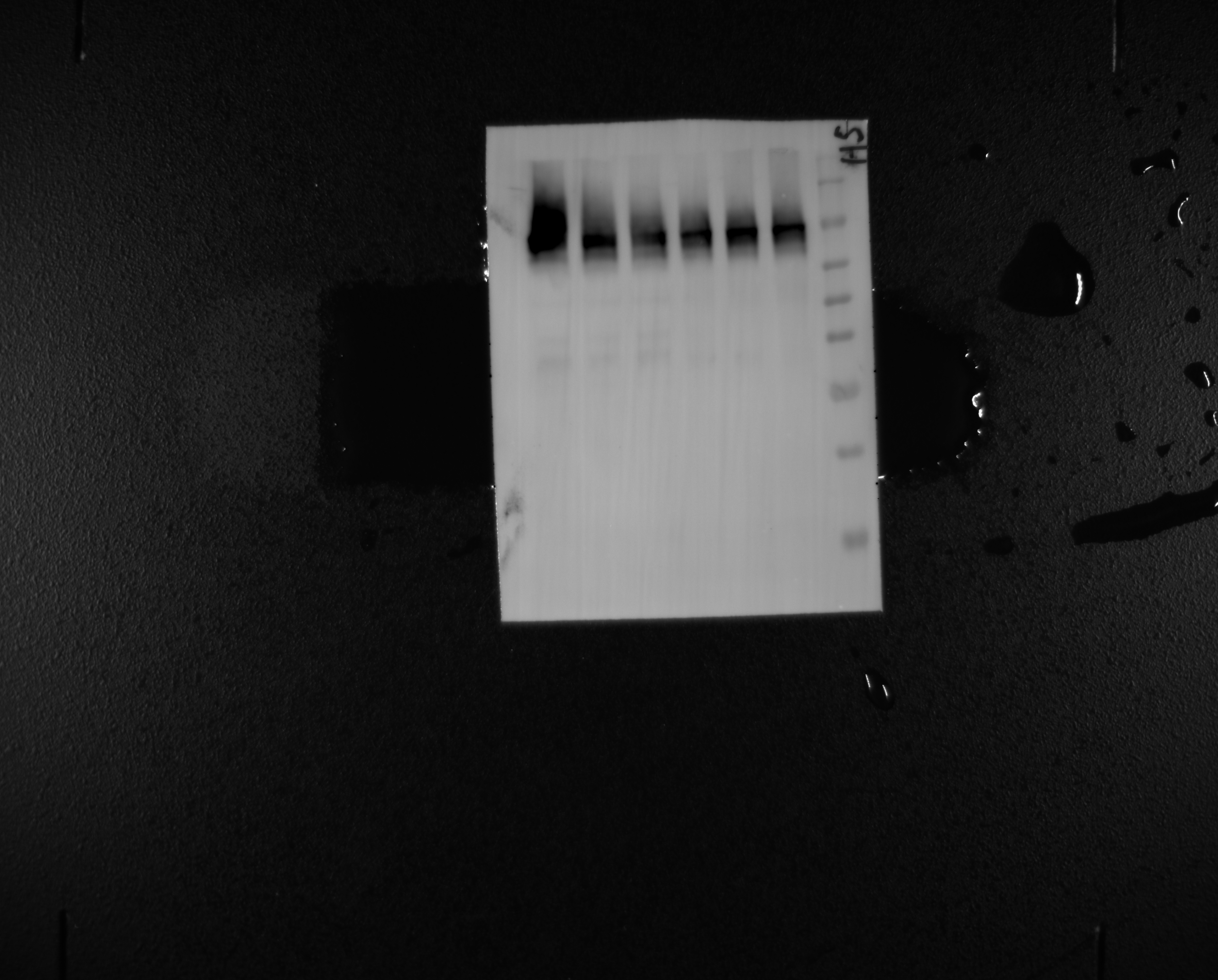 |
| LIAS | 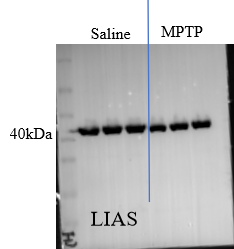 | 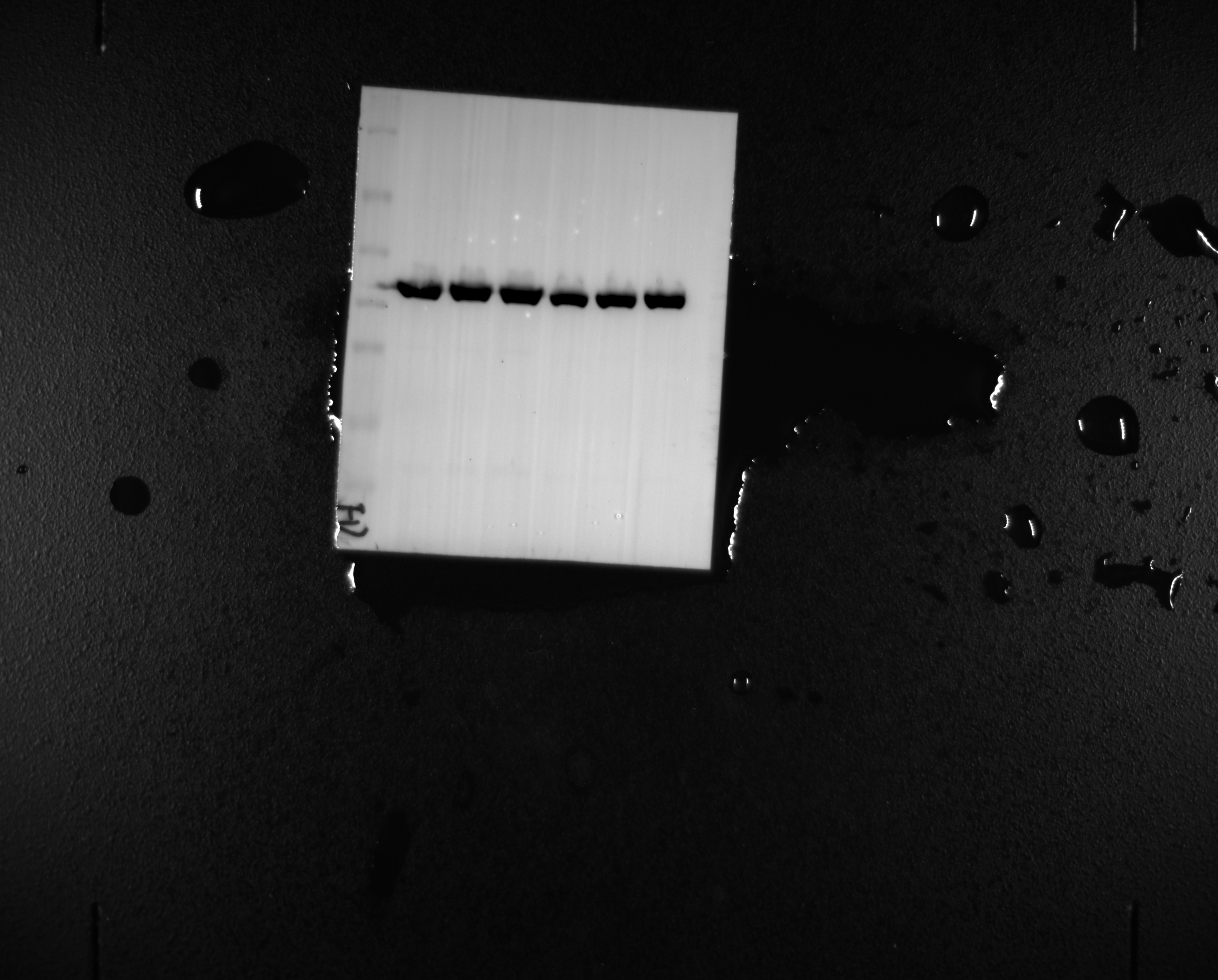 |
| β-actin | 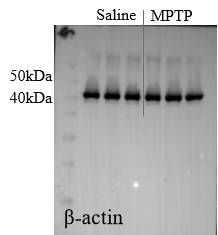 | 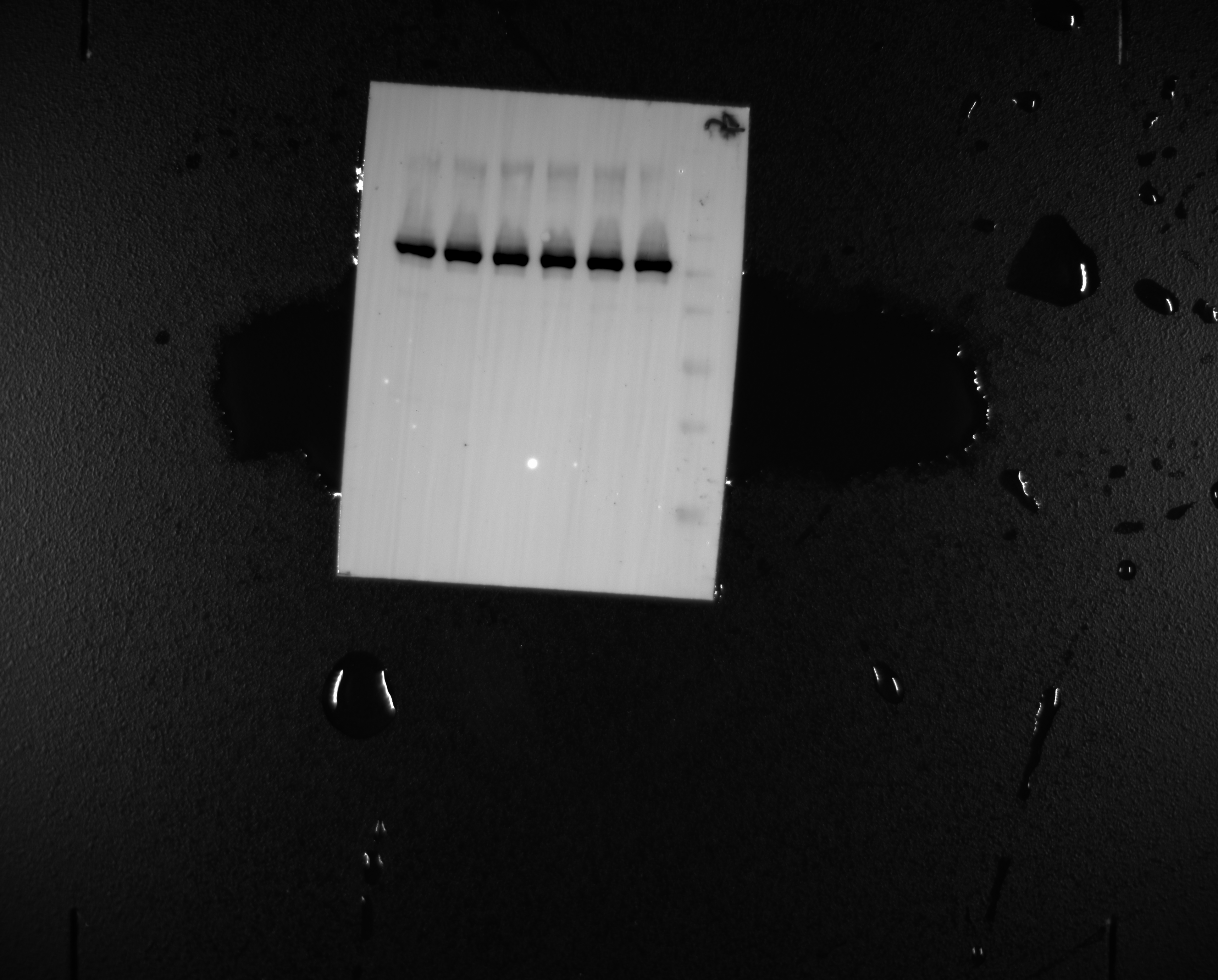 |
| LIP-DLAT DLST | 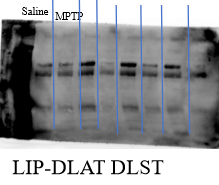 | 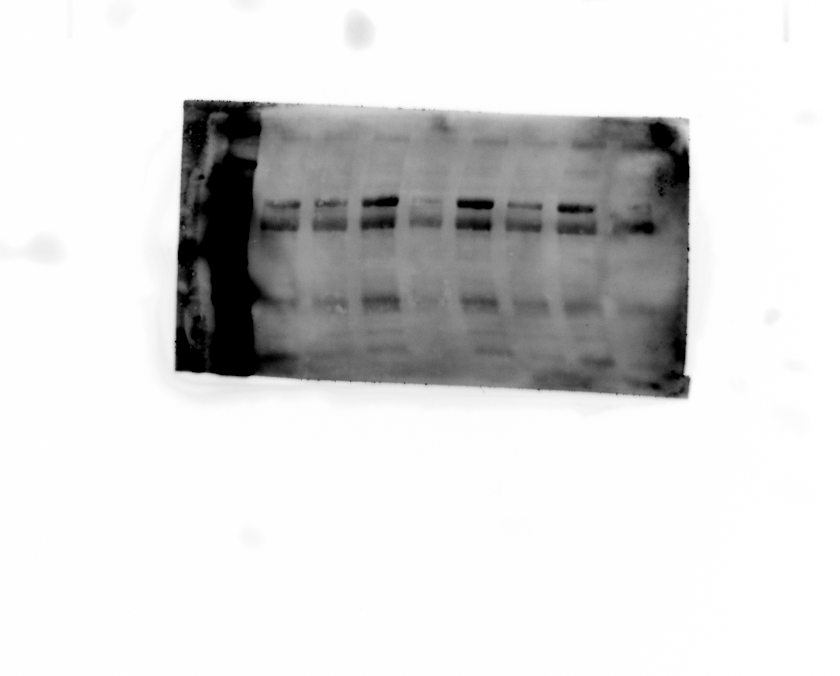 |
| β-actin | 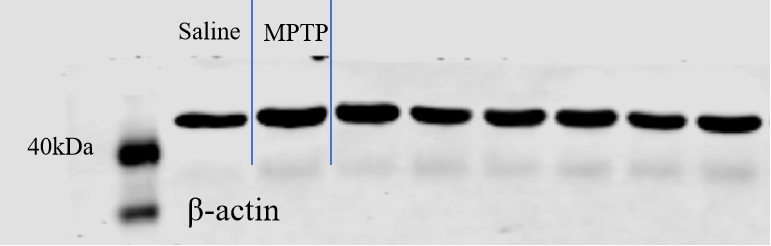 | 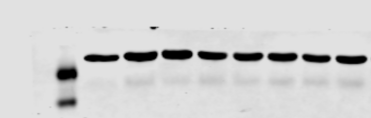 |
| DLAT oligomerization | 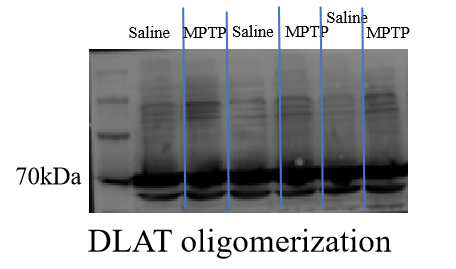 | 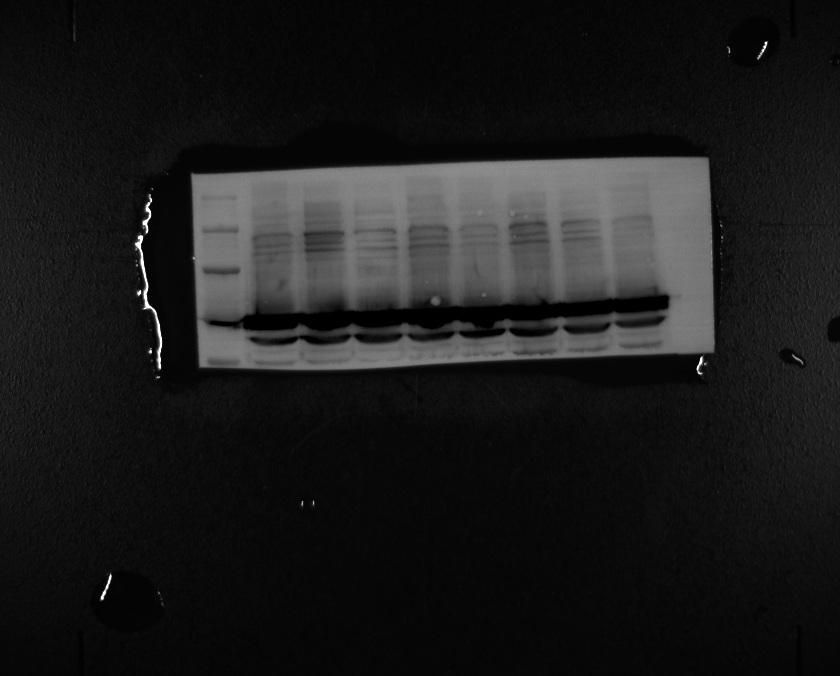 |
| β-actin | 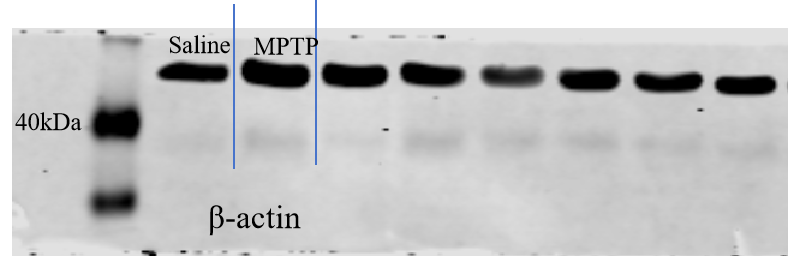 | 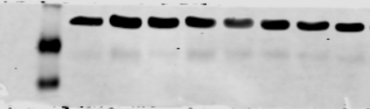 |

Supplement: S1 File — (DOCX) [file pone.0327550.s011.docx]
